# Host Engineering of Deep‐Blue‐Fluorescent Organic Light‐Emitting Diodes with High Operational Stability and Narrowband Emission

**DOI:** 10.1002/advs.202407278

**Published:** 2024-09-20

**Authors:** Wanqing Cai, Wansi Li, Xiaoge Song, Xiujie Zheng, Hao Guo, Chengwei Lin, Dezhi Yang, Dongge Ma, Maggie Ng, Man‐Chung Tang

**Affiliations:** ^1^ Faculty of Materials Science MSU‐BIT University Shenzhen 518172 China; ^2^ Institute of Materials Research Tsinghua Shenzhen International Graduate School Tsinghua University Shenzhen 518055 China; ^3^ State Key Laboratory of Luminescent Materials and Devices Institute of Polymer Optoelectronic Materials and Devices School of Materials Science and Engineering South China University of Technology 381 Wushan Road Guangzhou 510640 China

**Keywords:** blue OLED, BT.2020, operational stability, top‐emitting OLED, triplet–triplet up‐conversion

## Abstract

The realization of highly operationally stable blue organic light‐emitting diodes (OLEDs) is a challenge in both academia and industry. This paper describes the development of anthracene–dibenzofuran host materials, 2‐(10‐(naphthalen‐1‐yl)anthracen‐9‐yl)naphtho[2,3‐b]benzofuran (**Host 1**) and 2‐(10‐([1,1′‐biphenyl]‐2‐yl)anthracen‐9‐yl)naphtho[2,3‐b]benzofuran (**Host 2**), namely for use in the emissive layer of an OLED stack. A multiple‐resonance thermally activated delayed serves as the blue fluorescence emitter and exhibits an initial luminance of 1000 cd m^−2^ and long operational stability (i.e., time to decay to 90% of initial luminance) of 249 h. Furthermore, a deep‐blue OLED with an optimized top‐emitting architecture with a high current efficiency of 154.3 cd A^−1^, is fabricated and calibrated to a Commission International de l’Éclairage *y* chromaticity coordinate of 0.048. Moreover, the emission spectrum of this OLED has a narrowband peak at 476 nm with a full width at half maximum (FWHM) of 16 nm. This work provides valuable insights into the design of anthracene‐based host materials and highlights the importance of host optimization in improving the operational stability of OLEDs.

## Graphical abstract

This paper explores the demonstration of highly stable blue organic light‐emitting diodes (OLEDs) using novel anthracene–based host materials. Integrating a multiple‐resonance emitter, the OLEDs exhibit exceptional long‐operational stability (LT90 = 249 h). Additionally, a top‐emitting deep‐blue OLED with superior current efficiency and precise emission characteristics underscores the significance of host material optimization for enhancing OLED stability.

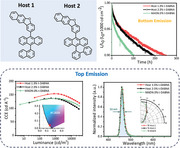

## 1 Introduction

Organic light‐emitting diodes (OLEDs) are widely applied in optoelectronic devices, such as televisions, monitors, smartphones, and smartwatches.^[^ ^1^, ^2^, ^3^, ^4^, ^5^ ^]^ However, while devices based on highly efficient red and green OLEDs with high operational stability have been fabricated, blue OLEDs face significant challenges, particularly regarding operational lifetime and electroluminescence performance. It has been well‐reported that bimolecular processes, namely triplet‐triplet annihilation (TTA), triplet‐polaron annihilation (TPA), and singlet‐polaron annihilation (SPA), are the primary degradation processes in OLEDs. These processes are responsible for forming high‐energy polarons that induce radical reactions in the emissive layer (EML) of an OLED.^[^ ^6^ ^]^ Several strategies to extend the lifetime of blue OLED devices have been explored, such as the employment of a stacked device structure with two emissive layers with graded dopant concentrations,^[^ ^7^ ^]^ the introduction of an excited state manager molecule as a sacrificial agent,^[^ ^8^ ^]^ and the incorporation of sky‐blue thermally activated delayed fluorescence (TADF) emitters as assistant dopants.^[^ ^5^ ^]^ However, despite these efforts, the operational stabilities of blue OLEDs are far from meeting the requirements for commercialization.

The long operational lifetimes of blue fluorescence OLEDs have sparked extensive research attention. However, these devices' maximum internal quantum efficiency (IQE) is limited to 25% due to the spin statistic.^[^ ^9^, ^10^, ^11^ ^]^ An effective method that allows a process to maintain a long‐lasting operational device lifetime with a comparatively high yield of exciton production is known as triplet‐triplet upconversion (TTU).^[^ ^11^ ^]^ In the production processes for blue OLEDs, it is observed that when two triplet excitons with similar energies (approximately 1.6 eV, such as anthracene derivatives) interact, they can combine to generate a single bright excited singlet exciton with energies around 3.2 eV. This approach permits the design of low‐triplet energy while maintaining superior exciton utilization efficiency. Theoretically, if all triplet states contribute to this process, the maximum expected IQE is estimated to reach 62.5%. While the energy level of the anthracene‐based derivative is typically in the order of S_1(emitter)_, T_2(host)_ < 2T_1(host)_ < Q_1(host)_, indicating that S_1_ and T_1_, but not Q_1_, are accessible via TTU.^[^ ^11^ ^]^ The total radiative exciton ratio is limited to 40% (singlet exciton, 25%; delayed contribution, 15%).^[^ ^11^ ^]^ Yet, a recent report showed that anthracene‐based materials with a small singlet‐triplet energy gap (Δ_EST_) (≈0.1−0.2 eV) might impede up‐conversion reverse intersystem crossing (RISC) from the T*_n_* states to the S_1_ state, thereby improving the exciton harvesting beyond 40%.^[^ ^12^ ^]^

Multi‐resonance TADF (MR‐TADF) emitters have narrowband emission and thus have been widely used in recently developed OLEDs.^[^ ^13^, ^14^, ^15^ ^]^ These materials incorporate electron‐withdrawing boron atoms and electron‐donating nitrogen atoms that induce complementary MR effects, leading to the separation of the highest occupied molecular orbital (HOMO) and lowest unoccupied molecular orbital (LUMO).^[^ ^16^ ^]^ Additionally, these configurations minimize structural relaxation and vibrational effects, thereby inducing a small Stokes shift and a narrow full width at half maximum (FWHM). However, the Δ_EST_ of MR‐TADF emitters is larger than those of conventional TADF emitters. Today, only a few MR‐TADF emitters with Commission International de l’Éclairage *y*‐coordinate (CIE*_y_*) of less than 0.1 have reported operation lifetimes for blue OLEDs. Kim and co‐workers^[^ ^17^ ^]^ reported high‐efficiency blue‐fluorescent OLEDs based on MR‐TADF emitters with CIE coordinates of (0.134, 0.131) and (0.137, 0.076), respectively. These devices exhibited maximum external quantum efficiencies (EQEs) of 10.2% and 8.6%, respectively, due to enhanced TTA processes and triplet‐harvesting efficiencies. Duan and co‐workers strategically introduced a mesitylboron locking unit into an MR emitter, resulting in a blue emission at 452 nm with an FWHM of 14 nm.^[^ ^18^ ^]^ Moreover, they achieved a remarkable time to decay to 97% of the initial luminance (LT_97_) of 178 h at a constant current density of 12 mA cm^−2^ in a TTA‐based device with a CIE*_y_* of 0.057. Nevertheless, to achieve the Broadcast Service Television 2020 (BT.2020) standard, a CIE*_y_* of less than 0.05 is highly desirable.^[^ ^19^ ^]^

**Figure 1:**
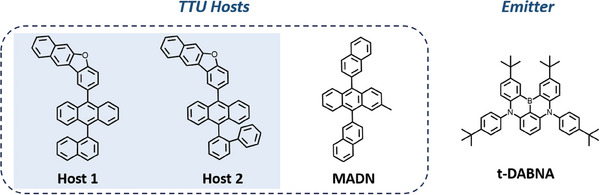
Molecular structures of TTU hosts and t‐DABNA.

In this study, we developed two anthracene‐based TTU host materials, namely **Host 1** and **Host 2** (**Figure** **1**). On the one hand, anthracene serves as a common TTU host material and blocking layer material. The anthracene moiety, with the presence of the low‐lying triplet state, can facilitate the triplet‐triplet up‐conversion process. Leveraging this foundational framework, the dibenzofuran moiety possesses high thermal stability, light extraction efficiency, and good charge transport properties. Simultaneously, its high triplet energy level leads to an elevated T_2_ state. We developed two TTU hosts by integrating the benzo(b)naphtho(2,3‐d)furan moiety onto the anthracene backbone. The electron‐transporting property of a benzo(b)naphtho(2,3‐d)furan moiety is anticipated to help balance hole and electron currents in the OLEDs and thus enhance their operational stability. In addition, we also compare the effect of the naphthalen‐1‐yl and 1,1′‐biphenyl‐2‐yl on the 9‐position of the anthracene to investigate the impact of π‐conjugation and steric hindrance on OLED stability. To realize efficient and stable deep‐blue‐fluorescent OLEDs, we employed t‐DABNA as the emitter. This choice is based on its S_1_ energy level (2.9 eV) being well‐matched to the S_1_ state of the hosts; such well‐matched energy alinement would ensure the energy transfer from the host to the emitter. An optimized device based on **Host 1** showed high operational stability, as shown by its decay time to 90% of the initial luminance (LT_90_) being up to 249 h at an initial luminance of 1000 cd m^−^^2^ with CIE coordinates of (0.125, 0.098). This represents a 2.5‐fold more operational stability than the corresponding commercially available MADN‐based OLED. These results demonstrate one of the longest operational lifetimes among blue OLEDs with a CIE*_y_* of less than 0.1. Furthermore, the device performance of the deep‐blue OLED with a CIE coordinate of (0.136, 0.048) that satisfies the requirement of BT.2020 was accomplished through a top‐emitting device architecture. This device exhibited a high current efficiency (calibrated to the CIE*_y_* coordinate) of 154.3 cd A^−1^, with an FWHM of 16 nm and an LT_90_ of 74.5 h at an initial luminance of 1000 cd m^−2^.

## 2 Results and Discussion

**Host 1** and **Host 2** were characterized by proton and carbon‐13 nuclear magnetic resonance spectroscopy and high‐resolution electrospray ionization mass spectrometry (Figures S1‐S6 in the Supporting Information). The thermal stabilities of **Host 1** and **Host 2** were determined by thermogravimetric analysis (TGA) and differential scanning calorimetry (DSC) (Figure S7, Supporting Information). **Host 1** and **Host 2** exhibited high decomposition temperatures (*T*_d_) exceeding 375 °C. Additionally, no glass transition temperatures (*T*_g_) were observed before decomposition, indicating that the compounds possess the necessary thermal stability for vacuum‐deposited OLEDs.

To investigate the electronic structures and the nature of the absorption and emission origins of **Host 1**, **Host 2**, and **MADN**, density functional theory (DFT) and time‐dependent density functional theory (TDDFT) calculations were performed. The first 15 singlet–singlet transitions of the host materials computed by TDDFT using the polarizable continuum model (PCM) are summarized in Table S2 (Supporting Information), and the simulated absorption spectra generated by Multiwfn^[^ ^20^ ^]^ are shown in Figure S8 (Supporting Information). The spatial plots of molecular orbitals (MOs) involved in the significant transitions are illustrated in Figures S9‐S11 (Supporting Information). For all the compounds, the low‐energy absorption band computed at ca. 400 nm was contributed by the S_0_→S_1_ transition attributed to the HOMO→LUMO excitation, where the HOMO and LUMO are the π and π* orbitals, respectively, localized on the anthracene moiety. Therefore, the low‐energy band can be assigned as a π→π* transition of the anthracene moiety. The orbital energy level diagram of **Host 1**, **Host 2**, and **MADN** is shown in Figure S12 (Supporting Information). **Host 1** and **Host 2** have similar HOMO energies (−5.49 and −5.45 eV, respectively), which were lower than that of **MADN** (−5.38 eV). Similarly, **Host 1** and **Host 2** have similar LUMO energies (−1.71 and −1.70 eV, respectively), which were lower than that of MADN (−1.63 eV). The trend of the computed HOMO and LUMO energies is consistent with that observed in the experiment. We further investigated the nature of the emissive state of the host materials, the geometries of the lowest‐lying singlet excited state (S_1_) of **Host 1**, **Host 2**, and **MADN** were optimized using the TDDFT method. Their emission wavelengths are summarized in Table S3 (Supporting Information), and the spatial plots of the singly occupied molecular orbitals (SOMOs) of the optimized S_1_ states are depicted in Figure S13 (Supporting Information). The emission wavelengths of **Host 1** and **Host 2** were ca. 478 nm, while that of **MADN** was red‐shifted to 503 nm. As both the lower singly occupied molecular orbital (LSOMO) and higher singly occupied molecular orbital (HSOMO) of each of these compounds are localized on the anthracene moiety, their emissions from the S_1_ state had the characteristics of the π→π* transition of the anthracene moiety.

The energy alignment of a material is a critical concern during the selection of host materials for TTA‐OLEDs, as it greatly influence on the maximum achievable radiative excitons generated from the TTA process. The energy level diagrams shown in Figures S14−S16 (Supporting Information) illustrate the energy alignments and hole and electron distributions of singlet and triplet excited states of the ground‐state geometries of the host materials. The energies of the T_2_ to T_4_ states in **Host 1** and **MADN** and the T_2_ to T_3_ states in **Host 2** were lower than the energies of their corresponding 2T_1_ states and thus were expected to have a maximum singlet exciton generated from a TTA of 15%.^[^ ^21^ ^]^ The RISC rate in a host material plays an important role in the degradation that results from TPA.^[^ ^22^ ^]^ Therefore, to gain insights into the RISC process from the triplet to the singlet excited state of the host materials, the RISC rate constants (*k*_RISC_) between the excited states were calculated. Moreover, the RISC processes between the S_1_ and T_4_ states in **Host 1** and **MADN** and between the S_1_ and T_3_ states in **Host 2** were potentially crucial to the photoluminescence quantum yields (PLQYs) and operational stabilities of the OLED devices fabricated in the present study, as the energy gaps between these singlet and triplet states are small. Thus, the corresponding energy differences (Δ_EST_), spin–orbit coupling (SOC) constants, and *k*_RISC_ were calculated and are summarized in Table S4 (Supporting Information). **Host 1** and **Host 2** have relatively higher *k*_RISC_ values than **MADN**, due to their higher Δ*E*(T_n_‒S_1_) and SOC constants. The order of *k*_RISC_ magnitude was **Host 1** > **Host 2** > **MADN**, which is consistent with the order of magnitude of PLQY and LT_90_ being **Host 1** > **Host 2** > **MADN**, suggesting that enhancing the efficiency of an RISC process in a host material may indirectly enhance the PLQY and the stability of the corresponding OLED device. This is probably due to the shortening of the triplet exciton lifetime, which would alleviate exciton annihilation processes and efficiency roll‐off in devices.^[^ ^23^, ^24^, ^25^, ^26^, ^27^, ^28^ ^]^

**Figure 2:**
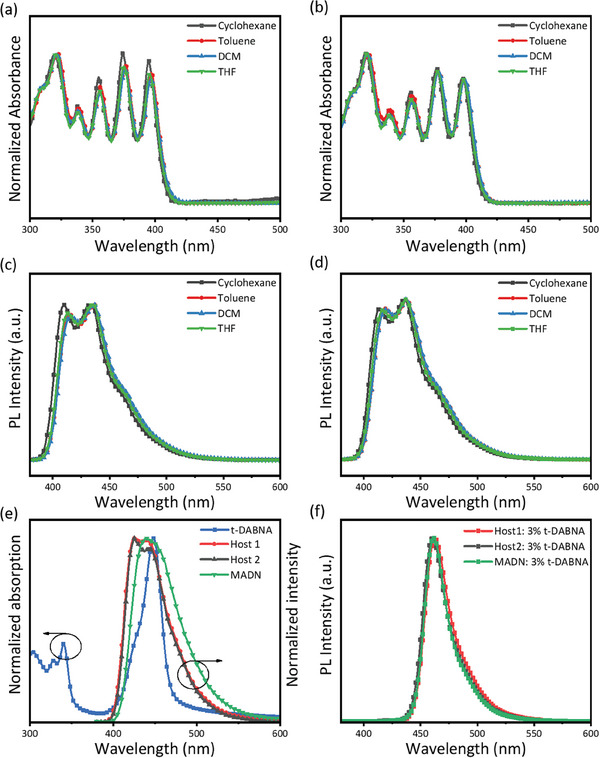
UV–vis absorption spectra of a) **Host 1** and b) **Host 2** in cyclohexane, toluene, DCM and THF. PL spectra of c) **Host 1** and d) **Host 2** in cyclohexane, toluene, DCM and THF. e) UV–vis absorption spectrum of t‐DABNA, and PL spectra of **Host 1, Host 2**, and MADN, as neat films. f) PL spectra of vacuum‐evaporated films of **Host 1, Host 2,** and MADN, respectively, doped with 3 wt% t‐DABNA (excited at 365 nm).

Next, regarding the electronic absorption and emission properties of **Host 1** and **Host 2**, we performed steady‐state ultraviolet–visible (UV–vis) absorption and emission spectroscopy to examine their photophysical properties. The UV–vis absorption spectra of **Host 1, Host 2** in cyclohexane, toluene, dichloromethane(DCM), and tetrahydrofuran (THF) solution are depicted in **Figure** **2**a,b. Notably, no discernible shift was observed in the absorption spectra, with increasing solvent polarity from cyclohexane to THF. The absorption bands at wavelengths longer than 350 nm are attributed to the spin‐allowed IL [π→π*] transitions of the dibenzofuran, naphthalene, and/or anthracene moieties. Upon excitation at λ ≥ 350 nm, the photoluminescence (PL) spectra of **Host 1** and **Host 2** in cyclohexane, toluene, DCM, and THF were analyzed (see Figure 2c,d). The emission behaviors of **Host 1** and **Host 2** in different solvents demonstrated similar characteristics, with a relatively broad FWHM of around 60 nm and peak at 414 and 436 nm. These emission bands were assigned to the IL [π→π* anthracene] excited state. While in the solid state thinfilm, **Host 1** and **Host 2** exhibited vibronically structured emission bands peaking at ca. 436 nm, while MADN showed a structureless emission band peaking at 440 nm (Figure 2e). The overlap between the emission spectra of the host materials and the absorption spectra of t‐DABNA suggests that efficient Förster energy transfer occurred in the films. Subsequently, t‐DABNA was introduced as a dopant into **Host 1** and **Host 2**, as well as MADN, at a concentration of 3 wt%. A distinct blue emission, centered at 462 nm with narrow emission bands (FWHM = 26 nm)as shown in Figure 2f. The transient PL of **Host 1** and **Host 2** is presented in Figure S17a,b (Supporting Information). The doped film displayed short fluorescent lifetimes ranging from 1 to 3 ns, as illustrated in Figure S17c (Supporting Information). The PLQYs of t‐DABNA doped into **Host 1**, **Host 2**, and **MADN** were 94.5%, 90.9%, and 87.6%, respectively. Furthermore, the preferred molecular orientations of t‐DABNA in these solid‐state thin films were determined by performing angular‐dependent photoluminescence (PL) measurements, as shown in Figure S18 (Supporting Information). The corresponding horizontal emitting dipole ratios (Θ) of **Host 1**, **Host 2**, and **MADN** were 0.82, 0.79, and 0.75, respectively. The selected photophysical data are summarized in **Table** **1**. Based on the equation of EQE = *η*_S/T_ × *γ* × *Φ*_PL_ × *η*_out_, where *η*_S/T_ is the ratio of radiative singlet/triplet excitons, *γ* denotes charge balance factor, *Φ*_PL_ represents the PL quantum yield, and *η*_out_ is the out‐coupling efficiency of emitted light. Thus, concerning the equation above, the fact that the value of Θ for t‐DABNA in **Host 1** was the highest indicates that the outcoupling efficiency of a device based on **Host 1**, would be higher than that of a device based on either **Host 2** or **MADN** if *γ* and *η*_S/T_ were of similar magnitude. This will be further discussed in the OLED section. Cyclic voltammetry studies have been performed on all host materials in DCM (0.1 m *^n^* Bu_4_NPF_6_). The compounds' oxidation scans are shown in Figure S19 (Supporting Information), and the data are listed in Table S14 (Supporting Information). The oxidation couples are assigned to the ligand‐center oxidation of anthracene moiety. This assignment is supported by the fact that incorporating the dibenzofuran or aryl moieties onto the 9‐position of the anthracene does not alter the oxidation potential, which is consistent with photophysical studies and the DFT calculations.

**Table 1:** Summary of photophysical properties of neat host films and t‐DABNA (3 wt%)‐doped host films.

Host	PL peak^a)^ [nm]	FHWM^a)^ [nm]	HOMO^b)^ [eV]	LUMO^c)^ [eV]	Films doped with 3 wt% t‐DABNA				
					PL peak [nm]	FWHM [nm]	PLQY [%]	Θ [%]	τ [ns]
**Host 1**	436	59	−5.83	−2.92	463	26	94.5	82	1.7
**Host 2**	436	57	−5.84	−2.95	462	26	90.9	79	2.7
**MADN**	440	64	−5.78	−2.90	462	26	87.6	75	1.0

^a)^ Measured in neat film samples at room temperature;

^b)^ Measured by ultraviolet photo‐electron spectroscopy (UPS);

^c)^ Estimated from HOMO energies and optical bandgaps.

**Figure 3:**
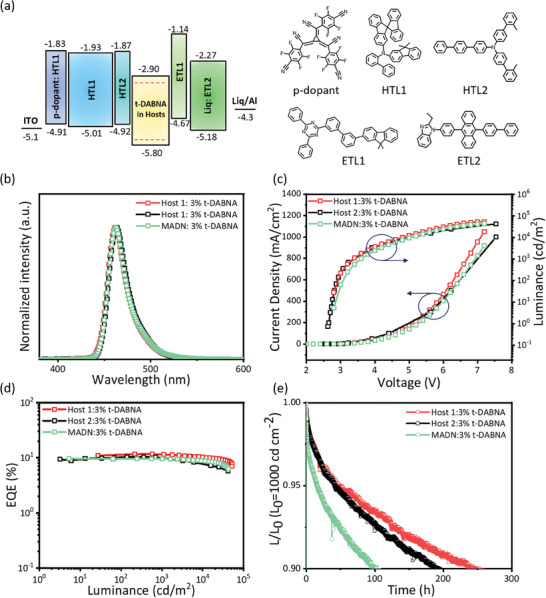
a) Left: schematic of structure of device showing components’ energy levels and thicknesses; right: molecular structures of materials used in the device. b) Current density–voltage–luminance characteristics, c) electroluminescence spectra, d) external quantum efficiency versus luminance, and e) operational lifetime with an initial luminance of 1000 cd m^−2^ of the devices composed of various hosts doped with 3 wt% t‐DABNA.

We then studied their HOMO levels and LUMO levels by UV–vis and ultraviolet photoelectron spectroscopy (UPS) (Figure S20, Supporting Information). The molecular structures of the materials employed in the devices, along with the device architecture, are depicted in **Figure** **3**a. The HOMO and LUMO energy levels of **Host 1**, **Host 2**, **MADN** and other materials employed in the OLED stack were estimated by combining the UPS results (Figures S21 and S22, Supporting Information) with the optical bandgaps obtained from UV–vis spectroscopy and are listed in Table 1 and Table S15 (Supporting Information). The HOMO energy levels of **Host 1** and **Host 2** were determined to be approximately −5.8 eV; the optical energy gaps were estimated to be 2.91 and 2.89 eV, respectively; and their LUMO energy levels were calculated to be −2.92 and −2.95 eV, respectively.

Host engineering towards OLED operational stability has been studied by commercially available OLED materials with the device architecture of ITO/3 wt% p‐dopant: HTL1 (10 nm)/HTL1 (50 nm)/HTL2 (5 nm)/EML (20 nm)/ETL1 (5 nm)/50 wt% Liq: ETL2 (30 nm)/Liq (2 nm)/aluminum (120 nm), where “ITO” = indium tin oxide, “HTL” = hole‐transport layer, “ETL” = electron‐transport layer, and “Liq” = 8‐hydroxyquinolinolatolithium. To enable efficient electron injection from the cathode, Liq (50 wt%) was doped into the 30 nm thick ETL2, while a p‐dopant (3 wt%) was doped into the 10 nm thick HTL1 to facilitate hole injection from the anode.^[^ ^29^, ^30^, ^31^ ^]^ The emitting dopant t‐DABNA was incorporated into the EML and the host materials, i.e., **Host 1**, **Host 2**, and **MADN**. The layer composition and thickness, except for that of the EML, were kept constant in all devices.

The TTU device based on **Host 1**, **Host 2**, and **MADN** containing 3 wt% of t‐DABNA as a dopant (the optimized concentration) emitted blue light with peak wavelengths of 463 nm, corresponding to the CIE coordinates of (0.125, 0.098), (0.124, 0.102), and (0.124, 0.102), respectively. The device characteristics and EL performances are given in Figure 3 and **Table** **2**. The EL spectra closely resembled the PL spectra of the emitter, i.e., t‐DABNA, confirming that the blue emission originated from t‐DABNA. The OLEDs based on **Host 1**, **Host 2**, and **MADN** exhibited maximum EQEs of 11.7%, 10.4%, and 9.7%, respectively. In addition, due to their structure, these devices demonstrated efficient injection of electrons and holes, as evidenced by their turn‐on voltages being less than 2.7 V, which is rather low and very similar in magnitude to the optical band gap of t‐DABNA. Compared with the **Host 2**‐ and **MADN**‐based devices, the **Host 1**‐based device exhibited higher performance due to its higher PLQY and Θ value. By utilizing these anthracene‐based analogs, we achieve long operational stability OLED of LT_90_ of 249 h and 192 h at an initial luminance of 1000 cd m^−2^ in encapsulated OLED devices based on **Host 1** and **Host 2**, respectively, which are 2.5‐ and 1.9‐fold higher than the LT_90_ of the **MADN**‐based device, i.e., 100.4 h. To the best of our knowledge, these results represent one of the longest operational lifetimes among blue OLEDs with CIE*_y_* less than 0.1. Detailed comparisons of operation stabilities based on t‐DABNA are given in Table S16 (Supporting Information).

**Table 2:** Summary of characteristics of devices.

Host:3% t‐DABNA	*V*_on_ ^a)^ [V]	*η*_ext_ [%] Max/100/1000/5000^b)^	*η*_c_ [cd A^−1^]	*η*_P_ [lm W^−1^]	*λ*_EL_ ^c)^ [nm]	CIE^c)^ (*x*, *y*)	LT_90_ ^d)^ [h]
**Host 1**	2.7	11.70/11.40/11.51/10.68	8.49	8.89	463	(0.125, 0.098)	249.0
**Host 2**	2.7	10.40/10.15/9.87/8.40	7.11	7.52	463	(0.124, 0.102)	192.3
**MADN**	2.6	9.60/9.55/9.55/9.11	7.26	8.15	463	(0.124, 0.102)	100.4

^a)^ Measured at a luminance of greater than 1 cd m^−2^;

^b)^ maximum efficiency/efficiency at a luminance of 100/1000/5000 cd m^−2^;

^c)^ measured at 10 mA cm^−2^;

^d)^ with respect to an initial luminance of 1000 cd m^−2^.

**Figure 4:**
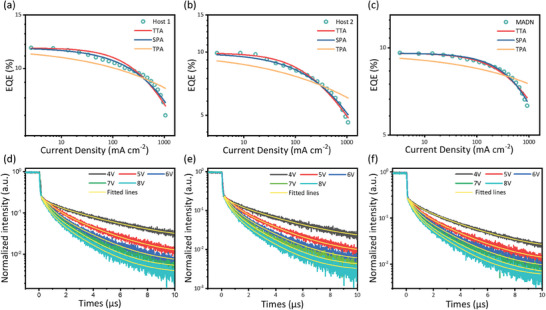
Efficiency roll‐off fitting results for external quantum efficiency–current density curves of a) **Host 1**‐, b) **Host 2**‐, and c) **MADN**‐based devices. Transient EL decay curves of d) **Host 1**‐, e) **Host 2**‐, and f) **MADN**‐based devices fitted with a TTA model (yellow lines). The dashed lines indicate the normalized pulse intensity at each voltage.

Furthermore, the **Host 1**‐based device exhibited suppressed efficiency roll‐off even at high luminance. For example, at a luminance of 1000 cd m^−2^ and 5000 cd m^−2^, the EQE values retained 11.51% and 10.68%, respectively, indicating that the efficiency roll‐off induced by the accumulation of triplet excitons was partially suppressed. To understand the mechanisms underlying this suppression of efficiency roll‐off in the devices, three models were considered: SPA, TPA, and TTA models. These models were used to fit the EQE versus current density curves of the devices (**Figure** **4**a–c) and analyzed the device behavior.^[^ ^25^, ^32^, ^33^, ^34^ ^]^ In the case of the **Host 1**‐based device, the EQE versus current density curves were fitted reasonably well by the SPA model at current densities of less than 300 mA cm^−2^. However, the EQE versus current density curves were not fitted well by any models at current densities exceeding 300 mA cm^−2^. This suggests other mechanisms might have contributed to the efficiency roll‐off at high current densities. In the case of the **Host 2**‐based device, the EQE versus current density curves were fitted well by the SPA model, indicating that SPA played a significant role in the efficiency roll‐off. However, at high current densities, i.e., greater than 150 mA cm^−2^, the EQE versus current density curves were fitted better by the TTA model, suggesting that TTA also contributed to the efficiency loss under these operating conditions. In contrast, in the case of the **MADN**‐based device, the EQE versus current density curves were fitted well by the TTA and SPA models. This may be attributable to the presence of many excitons and charge carriers in the current flow region, which would decrease the OLED's operational lifetime.

To validate the TTU mechanism, the voltage‐dependent EL emission decay curves were fitted with the TTU model, as shown in Figure 4d–f. As can be seen, these fittings demonstrated that the delayed emission observed in the EL decay curves originated from the TTU process. Additionally, the EL lifetimes of all three devices were found to be dependent on the applied voltage (Table S17, Supporting Information). That is, as the driving voltage was increased from 4 to 8 V, the devices’ delayed emission ratios exhibited a similar trend, with the largest ratio observed at a pulse intensity of 8 V. For example, the **Host 1**‐based device exhibited a delayed emission ratio of 36.73%. In contrast, those of the **Host 2** and **MADN**‐based devices were 33.8% and 31.92%, respectively. The **Host 1**‐based device exhibited the highest ratio, suggesting a well‐balanced exciton distribution and a larger collision rate within its EML than the other devices. Given that the radiative exciton ratio of delayed TTU emission equals *η*_S/T_ × TTU%, and that 25% of singlet excitons from hole–electron recombination equal *η*_S/T_, the *η*_S/T_ was determined to be approximately 36–39%. These values are very similar to the upper limits of *η*_S/T_, i.e., 40%, indicating that when TTU hosts have a state energy alignment of 2T_1_ > T_2_−T_3_, the RISC process from the T*_n_* states to the S_1_ state should also be taken into consideration. This is supported by the small Δ*E*(T_4_‒S_1_) of **Host 1** (0.06 eV) and **Host 2** (0.09 eV), as discussed in the TDDFT calculation section. Given that *η*_out_ is approximately 30% (due to its high Θ value), the value of *γ* is often assumed to be unity, and *Φ*_PL_ is approximately 87–94%, the theoretical EQEs (EQE = *η*_S/T_ × *γ* × *Φ*_PL_ × *η*_out_) were calculated to be approximately 9–11%, which is in excellent agreement with the experimental maximum EQEs of 11.4%, 10.4%, 9.7% for **Host 1**‐, **Host 2**‐, and **MADN**‐based devices, respectively.

**Figure 5:**
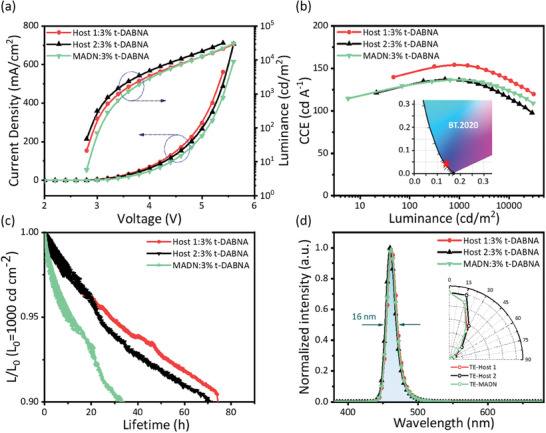
Performance of top‐emitting OLEDs composed of various hosts doped with 3 wt% t‐DABNA. a) Current density–voltage–luminance characteristics. b) CCE−luminance curves. c) Lifetimes measured at an initial luminance of 1000 cd m^−2^. d) EL spectra at a current density of 10 mA cm^−2^. Inset: Angle‐resolved luminance distribution.

Top‐emitting OLED (TEOLEDs) offer improved light‐extraction efficiencies, resulting in brighter displays. TEOLEDs also enhance color purity and accuracy due to reduced interference and light scattering.^[^ ^35^ ^]^ We fabricated TEOLEDs with a coupled optical cavity design that closely resembled conventional OLEDs’ design except for the two electrodes. The optical cavity consisted of a highly reflective anode made of silver (Ag: 120 nm) and a semi‐transparent cathode made of 10 wt% Mg:Ag (13 nm), where Mg is magnesium.^[^ ^36^ ^]^ The EL performances of the TEOLEDs are summarized in Table S18 (Supporting Information). As depicted in **Figure** **5**d, the TE‐**Host 1**, TE‐ **Host 2**, and TE‐**MADN** devices exhibited significantly narrower EL spectra and higher color purities than conventional devices. The FWHM in the EL spectra of these TEOLEDs were approximately 16 nm, almost half of the FWHM in the EL spectra of the conventional devices. As the current efficiency (in cd A^−1^) of blue OLEDs with a narrow FWHM is strongly influenced by the color coordinate, the current collection efficiency (CCE) was employed to evaluate the efficiency of these TEOLEDs.^[^ ^5^, ^37^, ^38^ ^]^ Thus, the current efficiency was divided by the CIE*_y_* color coordinate. The TE‐**Host 1**, TE‐**Host 2**, and TE‐**MADN** devices exhibited maximum EQEs of 15.1%, 13.2%, and 13.0%, respectively, corresponding CCEs of 154.3, 137.3, and 136.6 cd A^−1^. At a selected current density of 10 mA cm^−2^, the TE‐**Host 1** device demonstrated a low driving voltage of 3.36 V and CIE coordinates of (0.136, 0.048). The aforementioned CIE coordinates almost met the BT.2020 standard for CIE coordinates, i.e., (0.131, 0.046).^[^ ^39^ ^]^ Furthermore, at an initial luminance of 1000 cd m^−2^, an LT_90_ of 74.5 h was exhibited by the TE‐**Host 1** device, which was similar to that shown by the TE‐**Host 2** device, i.e., 72.0 h, and much longer than that exhibited by the TE‐**MADN** device, i.e., 33.3 h.

In conclusion, we conducted a comprehensive study of how two anthracene–based host materials, **Host 1** and **Host 2**, affect the photophysical behavior and performances of OLEDs. We found that although **Host 1** and **Host 2** had similar photophysical properties, they had different EL properties in devices. The order of *k*_RISC_, **Host 1** > **Host 2** > **MADN**, is consistent with PLQY and LT_90_ decreasing from **Host 1** to **Host 2** to **MADN**, suggesting that increasing the efficiency of the RISC process in a host material may indirectly enhance the PLQY and the stability of the corresponding OLED. Among the blue TTU‐OLEDs, a device based on **Host 1** exhibited superior stability, with an LT_90_ of 249 h at an initial luminance of 1000 cd m^−2^, approximately 2.5 times longer than the LT_90_ of a device based on **MADN**. In addition, a TE‐**Host 1** deep‐blue OLED demonstrated excellent performance, including a CCE of approximately 154.3 cd A^−1^, a CIE*_y_* coordinate of 0.048, and an FWHM of 16 nm. Overall, it is anticipated that this work provides the design principle of TTU host materials for improving their radiative exciton harvesting and further enhances their operationally stable blue OLEDs through the suppression of the unwanted TTA, TPA and SPA bimolecular processes.

## 3 Experimental Section

### Computational Details

Density functional theory (DFT) and time‐dependent density functional theory (TDDFT)^[^ ^40^, ^41^, ^42^ ^]^ calculations were performed with the Gaussian 16 suite of programs.^[^ ^43^ ^]^ The ground‐state (S_0_) geometries of **Host 1**, **Host 2**, and **MADN** were fully optimized in toluene using the PBE0 functional^[^ ^44^, ^45^, ^46^ ^]^ and the 6‐31G(d,p) basis set,^[^ ^47^, ^48^, ^49^ ^]^ in conjunction with the polarizable continuum model (PCM).^[^ ^50^, ^51^, ^52^ ^]^ The *k*_RISC_ values were computed by Molecular Material Property Prediction Package (MOMAP),^[^ ^53^ ^]^ whereas the SOC constants were computed by ORCA.^[^ ^54^, ^55^ ^]^ The Cartesian coordinates of the optimized geometries are given in Tables S5‒S13 (Supporting Information).

### Photophysical Measurements

UV–vis absorption spectroscopy was conducted on a Cary 500 UV–vis spectrophotometer (Agilent Technology) equipped with a xenon flash lamp. Steady‐state excitation and emission spectra were recorded on an FS5 spectrofluorometer (Edinburgh Instruments Ltd.). Photophysical measurements of vacuum‐deposited thin films were performed inside an optical cryostat (Optistat DN). The excited‐state lifetimes of thin films were measured using a conventional laser system. The excitation source was a laser (EPL‐375, Edinburgh Instruments) with a wavelength ranging from 369 to 381 nm and an output power (pulsed) of 0.11 to 0.15 mW. The luminescence decay signals were recorded on a photoluminescence spectrometer (FLS 980) and analyzed using an exponential fitting model. The absolute PLQYs in thin films were measured on an FS5 spectrofluorometer using an integrating sphere under nitrogen conditions.

### Cyclic Voltammetry Studies

Electrochemical measurements were carried out with a PalmSens4 electrochemical work station with platinum‐carbon as working electrode, platinum wire as the counter electrode, and a saturated calomel electrode (SCE) in saturated KCl aqueous solution as the reference electrode with a scan rate of 100 mV s^−1^.

### UPS

UPS was performed on an X‐ray photoelectron spectrometer (Thermo Fisher, ESCALAB Xi^+^) under a vacuum pressure of 8 × 10^−10^ Pa and with a power source energy of 21.22 eV (an He1 excitation source). The operating voltage was 12.5 kV, and 10 cycles were required to acquire a cumulative signal. The HOMO energy levels of the compounds in the films were determined by atmospheric ultraviolet photoelectron spectroscopy on a photoelectron emission spectrometer.

### Thermal Analysis Measurements

Thermogravimetry was performed using a Mettler TGA2 thermogravimeter and involved measuring the weight lost by a sample that was heated from 25 °C to 100 °C (where it was maintained for 15 min) and then to 800 °C at a rate of 10 °C min^−1^ under nitrogen. DSC was carried out in a SmartLab device (Rigaku) by heating samples from 50 °C to 350 °C at a rate of 10 °C min^−1^ and then cooling them at the same rate. The samples were heated in two different cycles.

### Device Fabrication and Measurement

OLEDs were fabricated by vacuum deposition without exposure to ambient air. Patterned ITO–glass substrates were cleaned ultrasonically and successively with detergent, acetone, deionized water, and isopropyl alcohol, and then dried at 65 °C in a baking oven. Subsequently, the substrates were treated with UV light and ozone for 30 min and then subjected to film deposition at a rate of 1.0–1.5 Å s^−1^ and a pressure of 3 × 10^−6^ Torr without a vacuum break. Next, the aluminum cathode was deposited at a rate of 4 Å s^−1^. The active area of each resulting OLED was 0.09 cm^2^. The thickness and deposition rate were monitored in situ during deposition using an oscillating quartz thickness monitor. Analyses of current density and luminance versus voltage measurements, EL spectra, and CIE coordinate measurements were performed using a source measurement unit (Keithley 2400) and a luminance color meter (CS‐200 Chroma Meter, Konica Minolta). EQEs were calculated based on the assumption of a Lambertian emission profile. In device lifetime tests, the luminance of the driving devices was measured using a luminance meter under a constant current density and with an initial luminance of 1000 cd m^−2^. The device structure is adapted from the reported literature.^[^ ^56^ ^]^

### Measurement of Transient EL

The EL spectra and transient EL curves were obtained on a spectrophotometer (PR650) and a spectrophotometer (FLS980, Edinburgh Instruments Ltd.), respectively. Additionally, transient EL was measured using a 100‐ or 200 µs electrical pulse at different current densities on a spectrophotometer (FLS980) and an oscilloscope.

## Conflict of Interest

The authors declare no conflict of interest.

## Author Contributions

W.C. and W.L. contributed equally to this work. W.C. and M.‐C.T. initiated and designed the research. M.‐C.T. designed the host compounds. W.C. and W.L. conducted the synthesis, characterization, and photophysical measurements of the compounds. W.L. carried out the OLED fabrication and characterizations. W.C., M.‐C.T., and W.L. wrote the manuscript. M.N. performed and analyzed the computational calculations. W.C. and M.‐C.T. supervised the work. All authors discussed the results and contributed to the manuscript.

## Supporting information



Supporting Information

## Data Availability

The data that support the findings of this study are available from the corresponding author upon reasonable request.
