# Supplementary material for: Host Engineering of Deep‐Blue‐Fluorescent Organic Light‐Emitting Diodes with High Operational Stability and Narrowband Emission
Source: Adv Sci (Weinh). 2024 Sep 20;11(43):2407278. doi: 10.1002/advs.202407278 (PMC11578301; doi:10.1002/advs.202407278)
Supplement: Supplementary file 1 — Supporting Information [file ADVS-11-2407278-s001.docx]

**Host Engineering of Deep-Blue-Fluorescent Organic Light-Emitting Diodes with High Operational Stability and Narrowband Emission**

Wanqing Cai^1,3‡*^, Wansi Li^2‡^, Xiaoge Song^2^, Xiujie Zheng^1^, Hao Guo^1^, Chengwei Lin^3^, Dezhi Yang^3^, Dongge Ma^3^, Maggie Ng^2^, and Man-Chung Tang^2*^

*^1^ Faculty of Materials Science, MSU-BIT University, Shenzhen 518172, China*

*^2^ Institute of Materials Research, Tsinghua Shenzhen International Graduate School, Tsinghua University, Shenzhen 518055, China*

*^3^ State Key Laboratory of Luminescent Materials and Devices, Institute of Polymer Optoelectronic Materials and Devices, School of Materials Science and Engineering, South China University of Technology, 381 Wushan Road, Guangzhou 510640, China*

^‡^ These authors contributed equally.

* Corresponding authors:

Email: wqcai@smbu.edu.cn; kobetang2021@sz.tsinghua.edu.cn

**Supporting Information**

Contents

[1. Characterizations 3](#_Toc164443301)

[2. Theoretical investigation 6](#_Toc164443302)

[3. Photophysical properties 29](#_Toc164443303)

[4. EQE-J curves fitting models 34](#_Toc164443304)

[5. Transient EL data 34](#_Toc164443305)

[6. Top-emitting device data 36](#_Toc164443306)

[References 37](#_Toc164443307)

## 1. Characterizations

**Host 1** and **Host 2** were synthesized according to the reported literature^[1]^. **Host 1**: ^1^H NMR (400 MHz, Chloroform-*d*) δ 8.41 (d, *J* = 5.7 Hz, 1H), 8.26 (m, Hz, 1H), 8.10 (d, *J* = 8.1 Hz, 1H), 8.03 (d, *J* = 7.8 Hz, 4H), 7.86–7.81 (m, 3H), 7.76–7.60 (m, 3H), 7.58–7.48 (m, 5H), 7.37–7.31 (m, 2H), 7.27–67.26 (m, 2H), 7.23–7.21 (m, 2H). ^13^C NMR (151 MHz, CDCl_3_) δ 157.14, 155.25, 136.94, 136.69, 136.67, 135.25, 133.66, 133.55, 133.14, 131.57, 130.63, 130.34, 130.19, 129.20, 128.45, 128.43, 128.26, 128.24, 128.14, 127.81, 127.12, 127.01, 126.62, 126.60, 126.30, 126.28, 126.02, 125.95, 125.62, 125.26, 125.22, 124.40, 124.24, 124.13, 124.12, 119.45, 111.54, 107.16. High-resolution ESI-MS m/z calcd for C_40_H_24_O 520.18, found 520.18.

**Host 2**: ^1^H NMR (400 MHz, DMSO-*d_6_*) δ 8.76 (d, *J* = 30.3 Hz, 1H), 8.39–8.28 (m, 1H), 8.25 (d, *J* = 3.1 Hz, 1H), 8.13 (m, 1H), 8.04 (t, *J* = 8.3 Hz, 1H), 7.96 (dd, J = 8.4, 3.2 Hz, 1H), 7.75–7.51 (m, 10H), 7.45–7.32 (m, 5H), 7.05–6.94 (m, 5H). ^13^C NMR (151 MHz, CDCl_3_) δ 157.04, 155.21, 143.11, 141.26, 141.19, 137.12, 136.53, 136.46, 133.54, 133.51, 133.09, 133.08, 132.52, 131.69, 131.52, 130.33, 130.15, 130.14, 130.07, 130.05, 128.47, 128.45, 128.43, 128.41, 128.20, 127.77, 127.46, 127.42, 127.29, 127.05, 127.04, 126.93, 126.49, 126.47, 125.89, 125.23, 125.06, 124.96, 124.34, 124.22, 124.11, 124.08, 124.02, 119.52, 119.39, 111.40, 111.34, 107.10, 107.07. High-resolution ESI-MS m/z calcd for C_42_H_26_O 546.20, found 546.20.

**Figure S1.** ^1^H NMR spectrum (400MHz, CDCl_3_) of **Host 1**.

**Figure S2.** ^1^H NMR spectrum (400MHz, DMSO-*d_6_*) of **Host 2**.

**Figure S3.** ^13^C NMR spectrum (101 MHz, CDCl_3_) of **Host 1**.

**Figure S4.** ^13^C NMR spectrum (101 MHz, CDCl_3_) of **Host 2**.


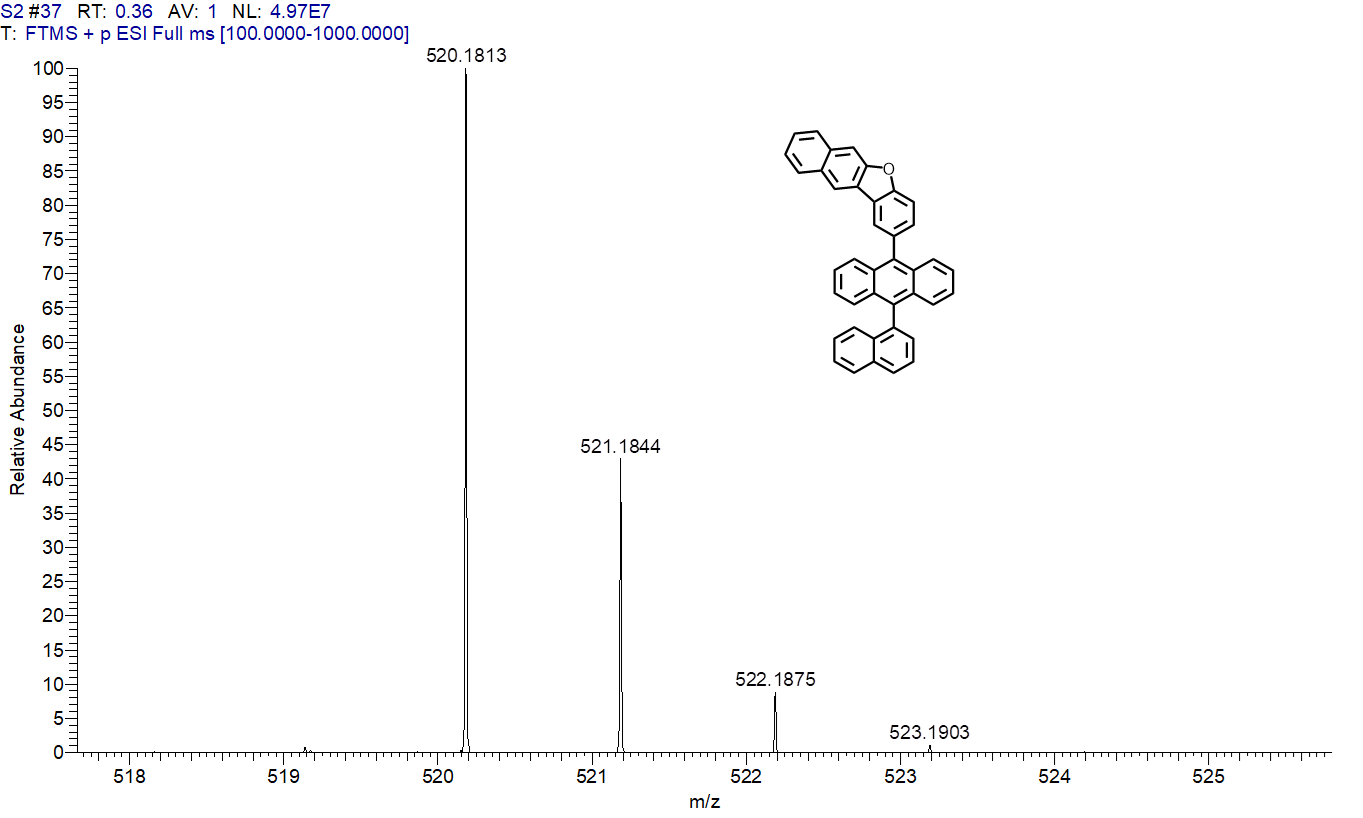


**Figure S5.** High-resolution ESI-MS of **Host 1**.


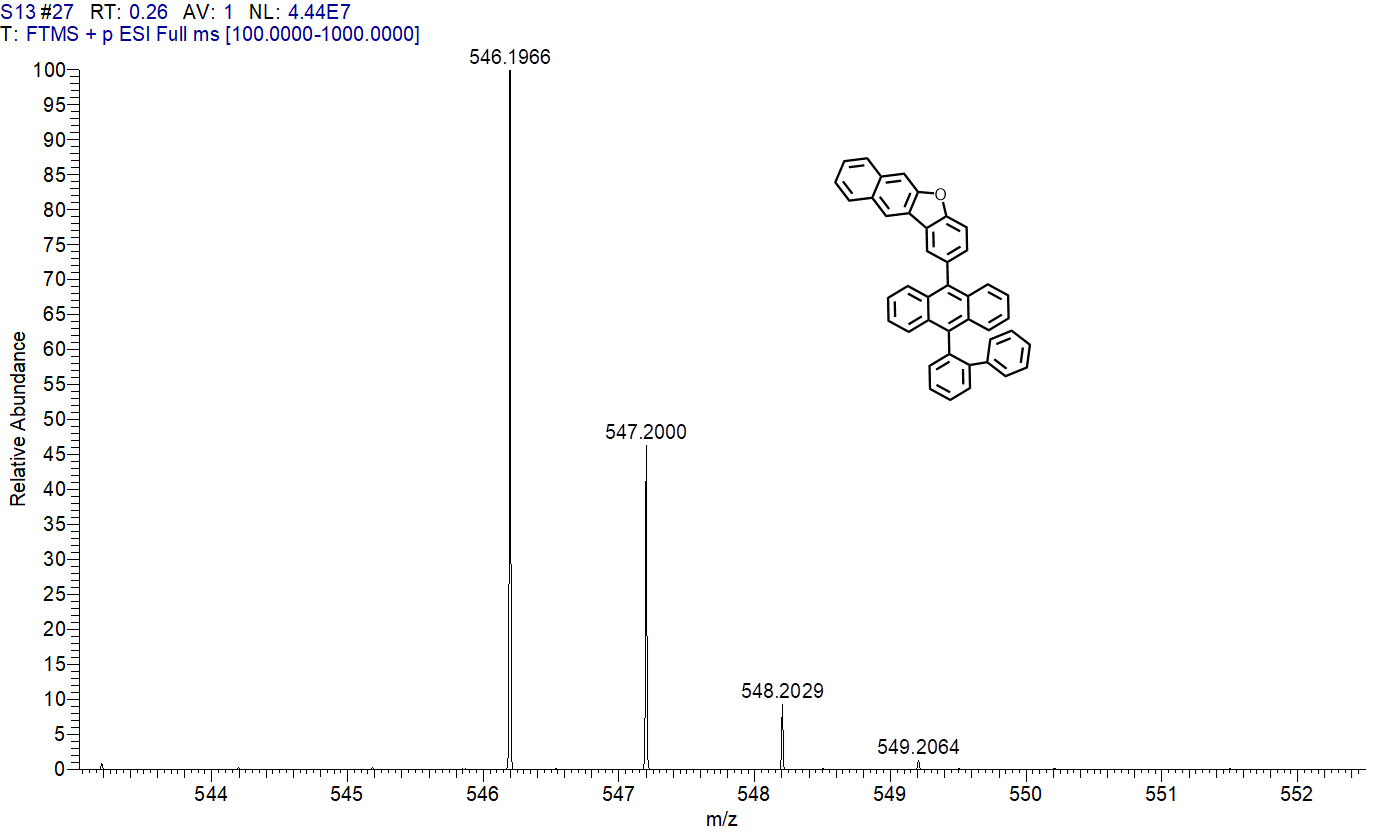


**Figure S6.** ESI-MS of **Host 2**.


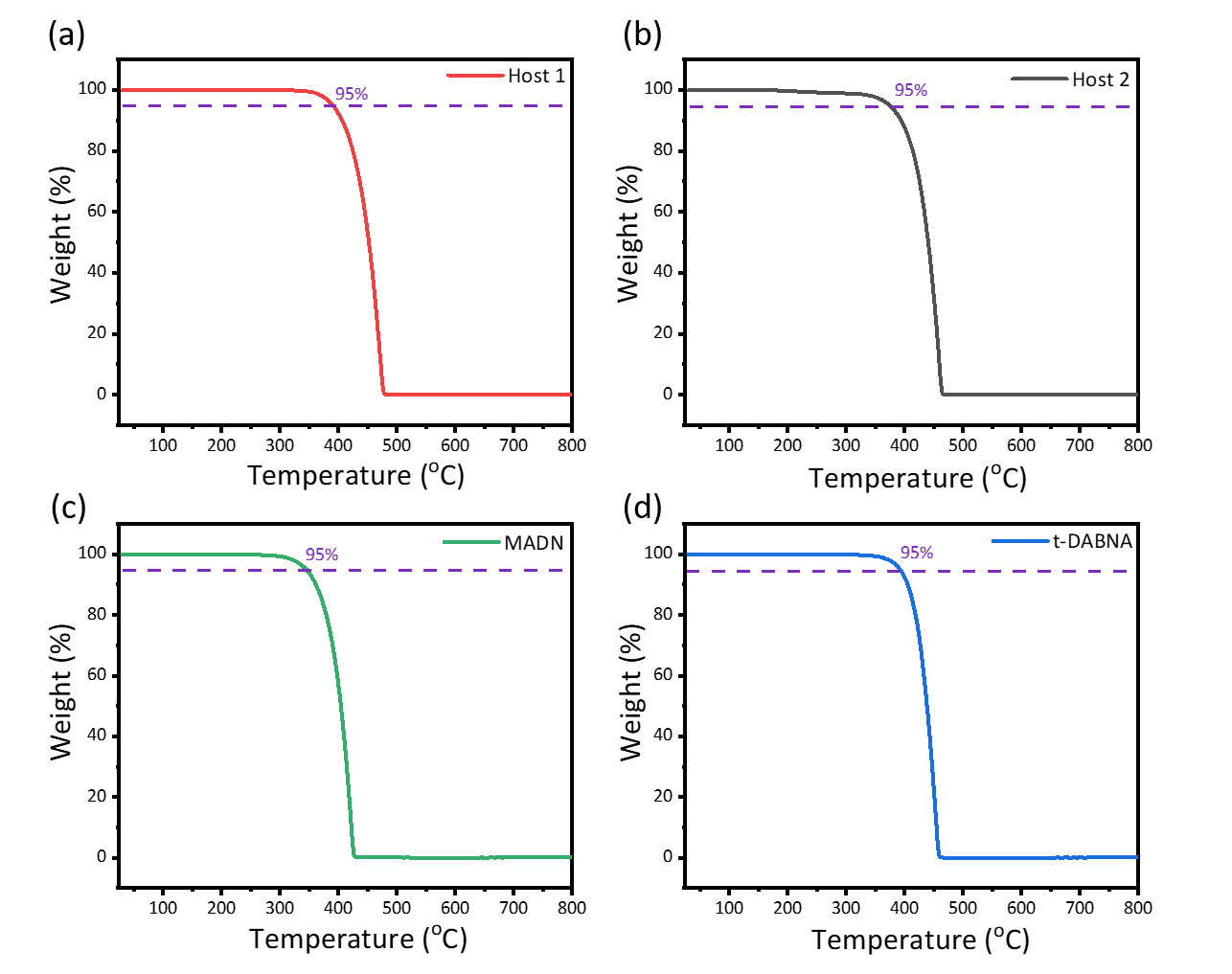


**Figure S7.** The thermogravimetric analysis (TGA) curves for of (a) **Host 1**, (b) **Host 2**, (c) **MADN** and (d) **t-DABNA**.

**Table S1.** Thermal properties of the blue host materials and emitter **t-DABNA**.

| Complex | *T_d_ ^a^*/℃ |
| --- | --- |
| **Host 1** | 390 |
| **Host 2** | 375 |
| **MADN** | 345 |
| **t-DABNA** | 392 |

*^a^* Decomposition temperature (*T****d***) is defined as the temperature at which the compound shows a 5 % weight loss.

## 2. Theoretical investigation

Singlet–singlet transitions in electronic spectra were computed using TDDFT at the same level based on optimised S_0_ geometries. To estimate the rates of RISC processes, TDDFT calculations at the same level were performed to optimise the geometries of the singlet and triplet excited states of **Host 1**, **Host 2**, and **MADN**. Vibrational frequency calculations were performed on the optimised geometries, and all stationary points were verified to be minima on the potential energy surface, as there were no imaginary frequencies observed (number of imaginary frequencies = 0).

**Table S2.** The first fifteen singlet excited states (S_n_) of **Host 1**, **Host 2** and **MADN** computed by TDDFT/PCM (toluene) at the PBE0 level based on the optimized ground-state (S_0_) geometries.

| Compound | S_n_ | Excitation*^a^*  (Coefficient)*^b^* | Vertical excitation wavelength (nm) | *f ^c^* |
| --- | --- | --- | --- | --- |
| **Host 1** | S_1_ | H→L (0.70) | 391 | 0.326 |
|  | S_2_ | H→L+1 (0.70) | 358 | 0.012 |
|  | S_3_ | H‒2→L (0.68) | 339 | 0.001 |
|  | S_4_ | H‒1→L (0.68) | 337 | 0.001 |
|  | S_5_ | H→L+2 (0.70) | 336 | 0.000 |
|  | S_6_ | H‒1→L+1 (0.68) | 326 | 0.109 |
|  | S_7_ | H‒4→L (0.45) | 314 | 0.000 |
|  |  | H→L+3 (‒0.38) |  |  |
|  | S_8_ | H‒3→L+1 (0.60) | 298 | 0.317 |
|  | S_9_ | H‒3→L (0.68) | 293 | 0.008 |
|  | S_10_ | H‒2→L+1 (0.70) | 285 | 0.001 |
|  | S_11_ | H‒2→L+2 (0.68) | 279 | 0.232 |
|  | S_12_ | H→L+4 (0.54) | 277 | 0.047 |
|  |  | H→L+3 (‒0.31) |  |  |
|  | S_13_ | H‒5→L (0.54) | 275 | 0.046 |
|  |  | H‒4→L (0.31) |  |  |
|  | S_14_ | H‒1→L+2 (0.66) | 272 | 0.002 |
|  | S_15_ | H‒5→L+2 (0.38) | 271 | 0.004 |
|  |  |  |  |  |
| **Host 2** | S_1_ | H→L (0.70) | 395 | 0.303 |
|  | S_2_ | H→L+1 (0.70) | 360 | 0.007 |
|  | S_3_ | H‒1→L (0.69) | 338 | 0.001 |
|  | S_4_ | H‒1→L+1 (0.68) | 327 | 0.115 |
|  | S_5_ | H‒4→L (0.43) | 318 | 0.001 |
|  |  | H→L+2 (0.43) |  |  |
|  | S_6_ | H‒3→L (0.70) | 307 | 0.002 |
|  | S_7_ | H→L+2 (0.52) | 301 | 0.019 |
|  | S_8_ | H‒2→L+1 (0.60) | 298 | 0.312 |
|  | S_9_ | H‒2→L (0.67) | 294 | 0.006 |
|  | S_10_ | H→L+3 (0.52) | 277 | 0.051 |
|  |  | H→L+4 (0.37) |  |  |
|  | S_11_ | H→L+5 (0.57) | 276 | 0.067 |
|  | S_12_ | H‒7→L (0.45) | 272 | 0.003 |
|  |  | H‒6→L (0.35) |  |  |
|  |  | H‒5→L (‒0.36) |  |  |
|  | S_13_ | H‒5→L (0.56) | 269 | 0.012 |
|  |  | H‒6→L (0.32) |  |  |
|  | S_14_ | H‒6→L (0.50) | 265 | 0.008 |
|  |  | H‒7→L (‒0.47) |  |  |
|  | S_15_ | H‒8→L (0.64) | 264 | 0.007 |
|  |  |  |  |  |
| **MADN** | S_1_ | H→L (0.70) | 395 | 0.338 |
|  | S_2_ | H→L+1 (0.66) | 340 | 0.009 |
|  | S_3_ | H→L+2 (0.68) | 338 | 0.003 |
|  | S_4_ | H‒1→L (0.62) | 321 | 0.005 |
|  | S_5_ | H‒2→L (0.66) | 320 | 0.007 |
|  | S_6_ | H‒3→L (0.46) | 314 | 0.002 |
|  |  | H→L+3 (0.41) |  |  |
|  | S_7_ | H→L+4 (0.67) | 284 | 0.020 |
|  | S_8_ | H→L+5 (0.49) | 279 | 0.011 |
|  |  | H→L+3 (0.37) |  |  |
|  | S_9_ | H‒2→L+1 (0.38) | 277 | 0.065 |
|  | S_10_ | H‒1→L+2 (0.49) | 277 | 0.018 |
|  | S_11_ | H‒4→L (0.37) | 274 | 0.160 |
|  | S_12_ | H‒5→L (0.35) | 273 | 0.040 |
|  |  | H‒4→L (‒0.31) |  |  |
|  | S_13_ | H‒4→L+2 (0.29) | 267 | 0.009 |
|  | S_14_ | H‒5→L (0.32) | 266 | 0.002 |
|  | S_15_ | H‒6→L (0.57) | 262 | 0.039 |

*^a^* The orbitals involved in the excitation (H = HOMO and L = LUMO).

*^b^* The coefficients in the configuration interaction (CI) expansion.

*^c^* Oscillator strengths.





**Figure S8.** Simulated UV-vis spectra of (a) **Host 1** (b)**Host 2** and (c) **MADN** computed by TDDFT/PCM in toluene.


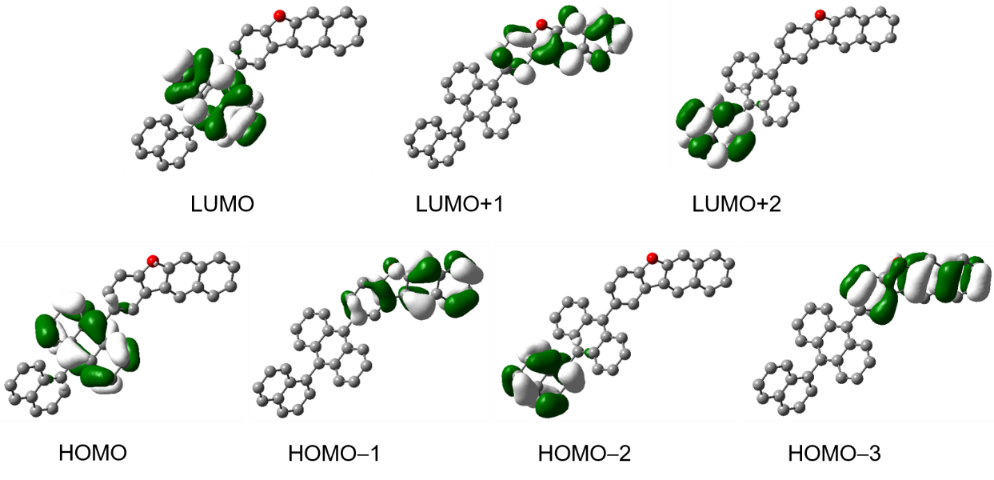


**Figure S9.** Spatial plots (isovalue = 0.03) of selected molecular orbitals of **Host 1** at the S_0_ geometry optimized at the PBE0 level. Hydrogen atoms are omitted for clarity.


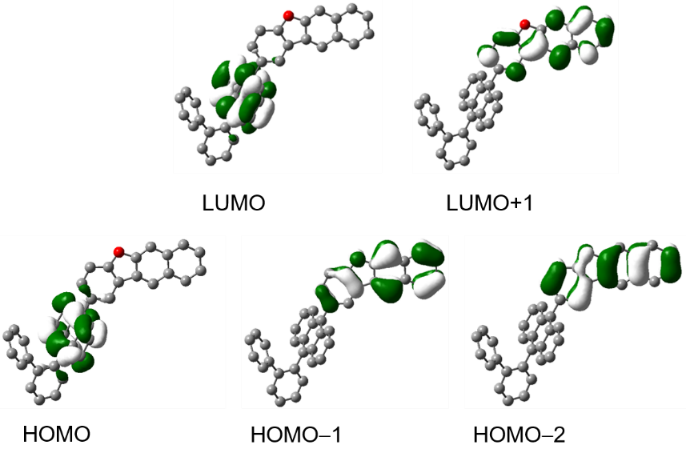


**Figure S10.** Spatial plots (isovalue = 0.03) of selected molecular orbitals of **Host 2** at the S_0_ geometry optimized at the PBE0 level. Hydrogen atoms are omitted for clarity.


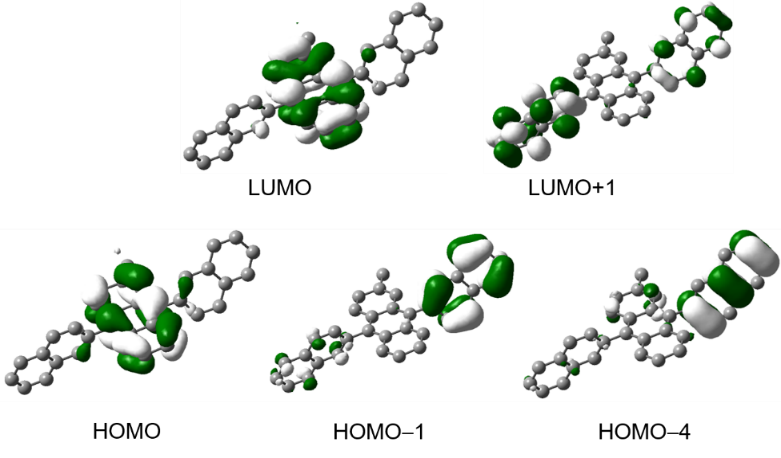


**Figure S11.** Spatial plots (isovalue = 0.03) of selected molecular orbitals of **MADN** at the S_0_ geometry optimized at the PBE0 level. Hydrogen atoms are omitted for clarity.


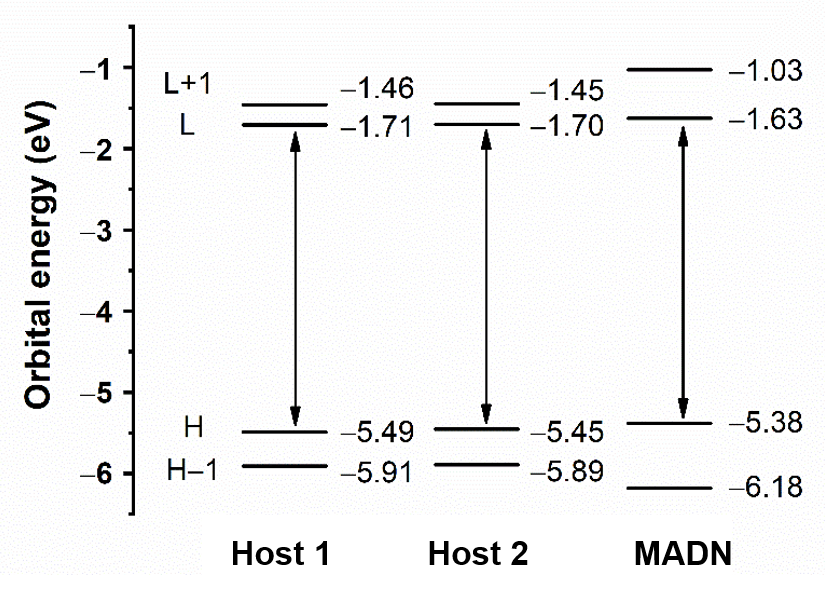


**Figure S12.** Orbital energy level diagram (H = HOMO and L = LUMO) of **Host 1**, **Host 2** and **MADN** optimized at the PBE0 level.

**Table S3.** Emission wavelengths of the optimized S_1_ states of **BH02**, **BH13** and **MADN**.

| Compound | Emission wavelength / cm^−1^ (λ / nm) |
| --- | --- |
| **Host 1** | 20988 (476) |
| **Host 2** | 20930 (478) |
| **MADN** | 19886 (503) |


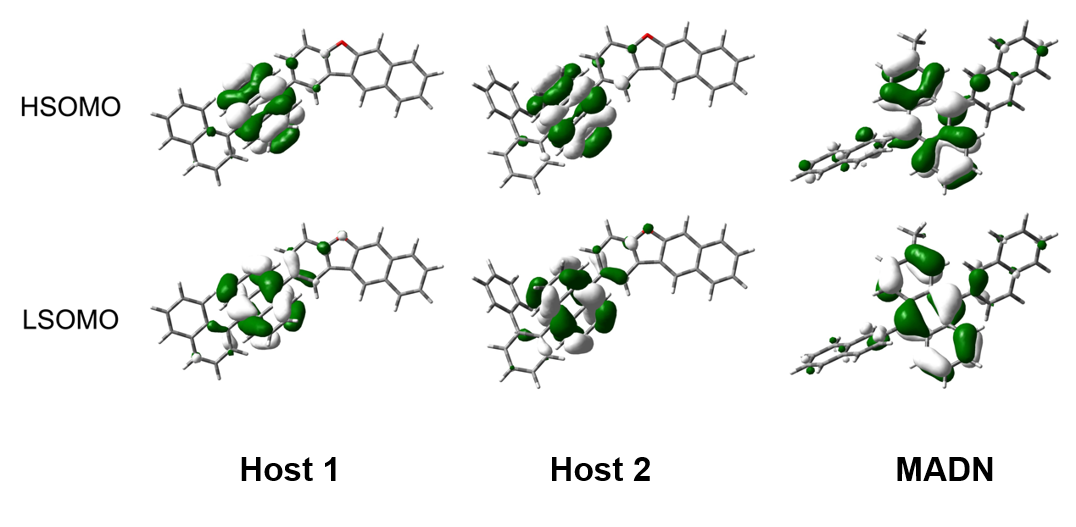


**Figure S13.** Spatial plots (isovalue = 0.03) of the SOMOs of **Host 1**, **Host 2** and **MADN** at the optimized S_1_ geometry.


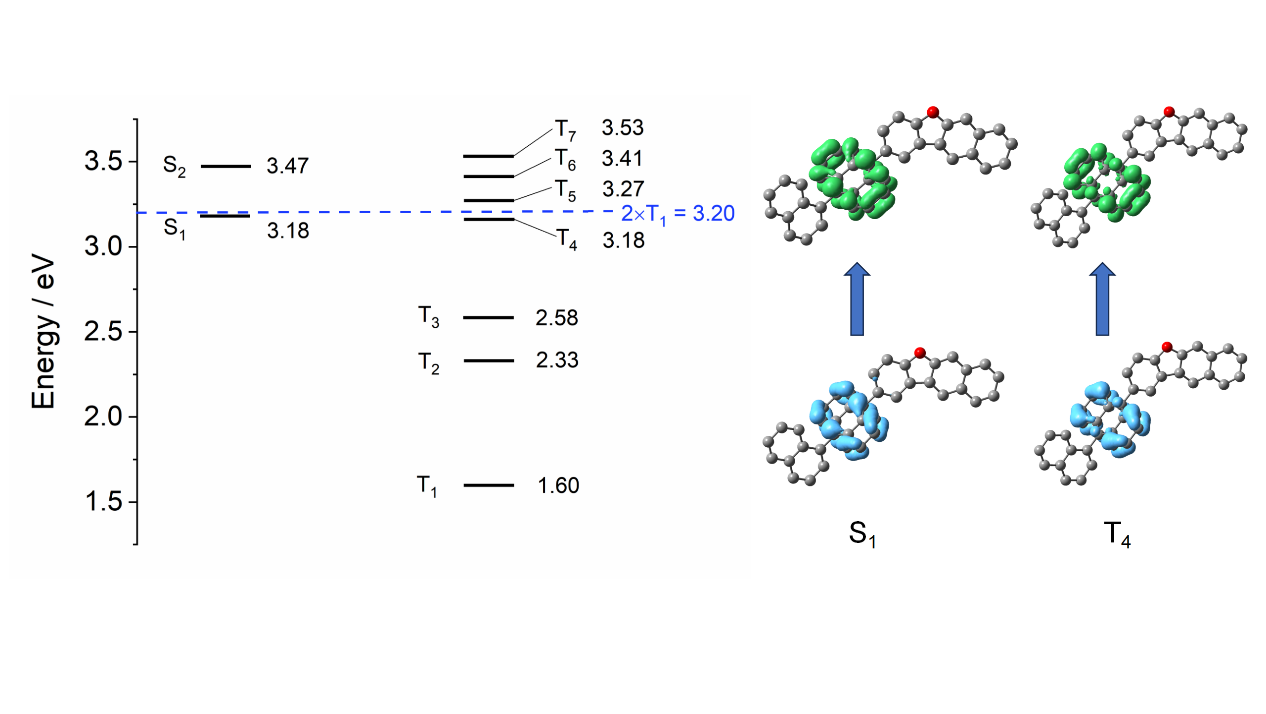


**Figure S14.** Energy level diagram showing the singlet and triplet excited states, and the hole (blue) and electron (green) distribution (isovalue = 0.002) corresponding to the S_1_ and T_4_ states of **Host 1** computed at the PBE0 level.


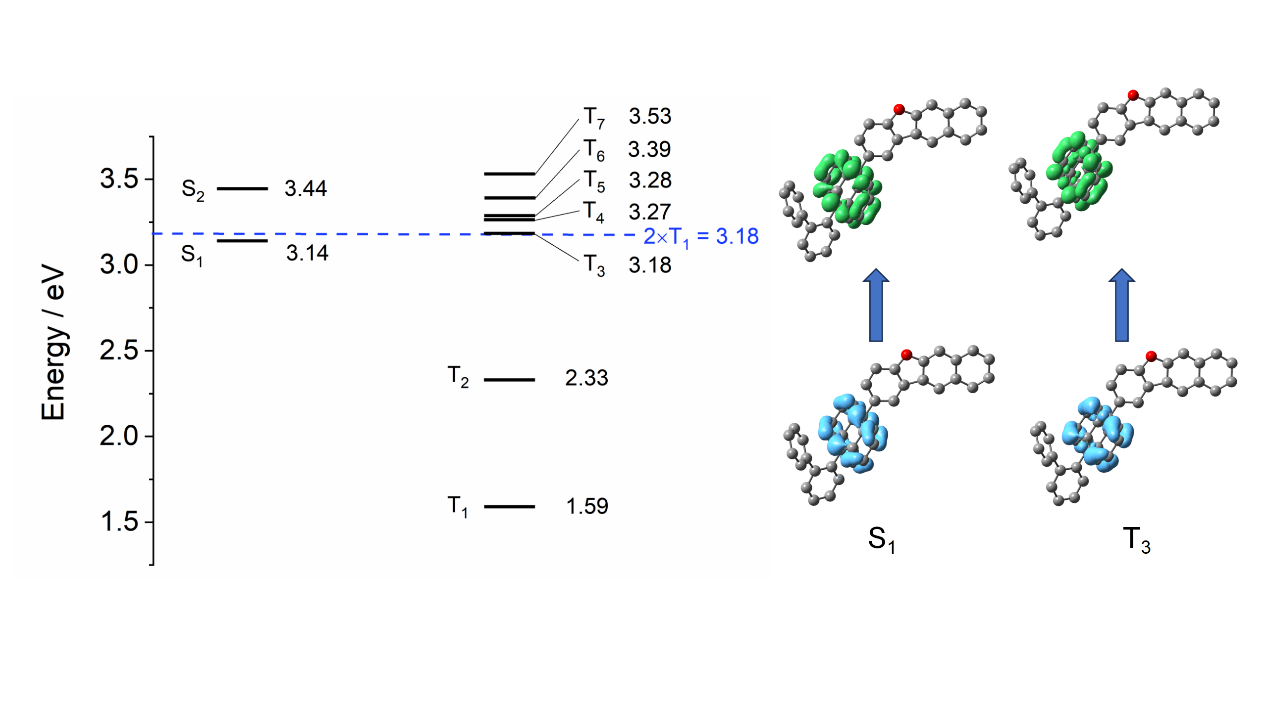


**Figure S15.** Energy level diagram showing the singlet and triplet excited states, and the hole (blue) and electron (green) distribution (isovalue = 0.002) corresponding to the S_1_ and T_3_ states of **Host 2** computed at the PBE0 level.


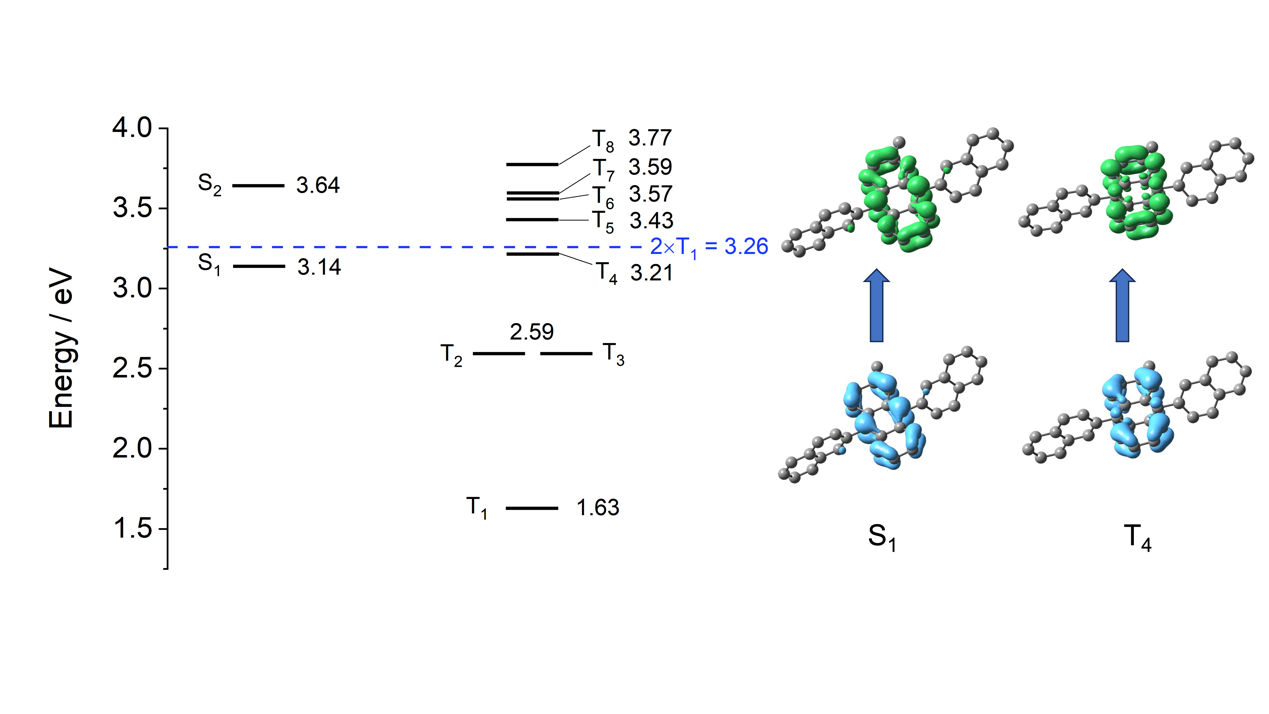


**Figure S16.** Energy level diagram showing the singlet and triplet excited states, and the hole (blue) and electron (green) distribution (isovalue = 0.002) corresponding to the S_1_ and T_4_ states of **MADN** computed at the PBE0 level.

**Table S4.** Computed energy differences (Δ*E*(T_n_‒S_1_)), SOC constants and *k*_RISC_ between the S_1_ and T_n_ states of **Host 1, Host 2** and **MADN**.

| Compound | **Host 1** | **Host 2** | **MADN** |
| --- | --- | --- | --- |
|  | S_1_-T_4_ | S_1_-T_3_ | S_1_-T_4_ |
| Δ*E*(T_n_‒S_1_) (eV) | 0.056 | 0.086 | 0.152 |
| SOC constant (cm^‒1^) | 0.176 | 0.178 | 0.100 |
| *k*_RISC_ (s^‒1^) | 1.64×10^6^ | 1.35×10^6^ | 6.42×10^5^ |

**Table S5.** Cartesian coordinates of the optimized S_0_ geometry of **Host 1**.

Energy = ‒1612.17033707 Hartrees

| 1 | C | 0.873037 | 2.615450 | 2.320616 |  | 44 | C | -8.837667 | 0.306214 | -0.163122 |
| --- | --- | --- | --- | --- | --- | --- | --- | --- | --- | --- |
| 2 | C | 0.315943 | 1.495462 | 1.771019 |  | 45 | H | -6.514825 | 2.710030 | -2.142298 |
| 3 | C | 1.034350 | 0.691001 | 0.833507 |  | 46 | H | -8.965791 | 2.954536 | -2.284356 |
| 4 | C | 2.378192 | 1.080501 | 0.490395 |  | 47 | H | -10.451246 | 1.412059 | -1.014145 |
| 5 | C | 2.918836 | 2.260939 | 1.086307 |  | 48 | H | -9.485913 | -0.367190 | 0.391559 |
| 6 | C | 2.191764 | 3.006490 | 1.970303 |  | 49 | C | 6.023535 | 1.943834 | -2.232167 |
| 7 | C | 0.469388 | -0.465981 | 0.261819 |  | 50 | C | 7.114113 | 1.522836 | -1.514131 |
| 8 | C | 3.130681 | 0.299941 | -0.406967 |  | 51 | C | 6.952574 | 0.674930 | -0.390517 |
| 9 | C | 2.570577 | -0.862347 | -0.969259 |  | 52 | C | 5.639584 | 0.259425 | -0.008290 |
| 10 | C | 1.222128 | -1.245652 | -0.638069 |  | 53 | C | 4.518131 | 0.710605 | -0.771304 |
| 11 | C | 0.680082 | -2.414284 | -1.256597 |  | 54 | C | 4.725524 | 1.535006 | -1.857689 |
| 12 | C | 1.420448 | -3.168584 | -2.122440 |  | 55 | H | 9.059515 | 0.547694 | 0.068697 |
| 13 | C | 2.756235 | -2.801577 | -2.431776 |  | 56 | H | 6.151074 | 2.593585 | -3.092991 |
| 14 | C | 3.309146 | -1.683017 | -1.875764 |  | 57 | H | 8.117005 | 1.833043 | -1.796360 |
| 15 | H | 0.307184 | 3.209165 | 3.032664 |  | 58 | C | 8.064401 | 0.226672 | 0.366348 |
| 16 | H | 3.928779 | 2.556585 | 0.821981 |  | 59 | C | 5.497343 | -0.589683 | 1.118935 |
| 17 | H | 2.623861 | 3.899466 | 2.412454 |  | 60 | H | 3.869194 | 1.874067 | -2.433911 |
| 18 | H | 0.985540 | -4.052082 | -2.580679 |  | 61 | C | 6.595673 | -1.006294 | 1.830884 |
| 19 | H | 3.336728 | -3.411130 | -3.118130 |  | 62 | C | 7.892783 | -0.595085 | 1.452480 |
| 20 | H | 4.326987 | -1.396728 | -2.119247 |  | 63 | H | 4.501318 | -0.907034 | 1.412024 |
| 21 | C | -0.923557 | -0.866261 | 0.609646 |  | 64 | H | 6.467823 | -1.656426 | 2.691491 |
| 22 | C | -1.148279 | -1.947698 | 1.481370 |  | 65 | H | 8.753084 | -0.931392 | 2.023810 |
| 23 | C | -2.430377 | -2.357741 | 1.836791 |  |  |  |  |  |  |
| 24 | C | -3.485223 | -1.648289 | 1.289935 |  |  |  |  |  |  |
| 25 | C | -3.304102 | -0.565330 | 0.417658 |  |  |  |  |  |  |
| 26 | C | -2.011769 | -0.171737 | 0.074978 |  |  |  |  |  |  |
| 27 | C | -5.512214 | -0.980427 | 0.796140 |  |  |  |  |  |  |
| 28 | C | -6.874454 | -0.886251 | 0.749928 |  |  |  |  |  |  |
| 29 | C | -7.432889 | 0.135663 | -0.057491 |  |  |  |  |  |  |
| 30 | C | -6.575162 | 1.025431 | -0.790022 |  |  |  |  |  |  |
| 31 | C | -5.170528 | 0.882463 | -0.705760 |  |  |  |  |  |  |
| 32 | C | -4.640096 | -0.114104 | 0.082484 |  |  |  |  |  |  |
| 33 | H | -7.510853 | -1.565024 | 1.308149 |  |  |  |  |  |  |
| 34 | H | -4.529704 | 1.560021 | -1.263398 |  |  |  |  |  |  |
| 35 | O | -4.808800 | -1.904939 | 1.523285 |  |  |  |  |  |  |
| 36 | H | -0.290659 | -2.474608 | 1.888995 |  |  |  |  |  |  |
| 37 | H | -0.689879 | 1.196926 | 2.046274 |  |  |  |  |  |  |
| 38 | H | -0.342515 | -2.694801 | -1.027865 |  |  |  |  |  |  |
| 39 | H | -2.598568 | -3.190189 | 2.511120 |  |  |  |  |  |  |
| 40 | H | -1.843076 | 0.662279 | -0.599987 |  |  |  |  |  |  |
| 41 | C | -7.167382 | 2.038793 | -1.589669 |  |  |  |  |  |  |
| 42 | C | -8.529851 | 2.173986 | -1.667988 |  |  |  |  |  |  |
| 43 | C | -9.373362 | 1.297480 | -0.946210 |  |  |  |  |  |  |

**Table S6.** Cartesian coordinates of the optimized S_1_ geometry of **Host 1**.

Energy = ‒1612.06453774 Hartrees

| 1 | C | -0.528954 | -2.349421 | -2.694913 |  | 44 | C | 6.945719 | 3.367344 | -5.815755 |
| --- | --- | --- | --- | --- | --- | --- | --- | --- | --- | --- |
| 2 | C | -0.121573 | -1.134022 | -2.130132 |  | 45 | H | 3.654285 | 2.259152 | -7.556471 |
| 3 | C | -1.013744 | -0.285782 | -1.448464 |  | 46 | H | 5.621795 | 2.888894 | -8.905865 |
| 4 | C | -2.370891 | -0.734952 | -1.275257 |  | 47 | H | 7.729344 | 3.598374 | -7.786777 |
| 5 | C | -2.739168 | -1.981371 | -1.819317 |  | 48 | H | 7.865670 | 3.677237 | -5.326938 |
| 6 | C | -1.840445 | -2.771295 | -2.544301 |  | 49 | C | -6.864895 | -0.815297 | -1.344560 |
| 7 | C | -0.595382 | 0.951826 | -0.854794 |  | 50 | C | -7.394093 | -1.179142 | -0.129368 |
| 8 | C | -3.281361 | 0.040326 | -0.493592 |  | 51 | C | -6.580451 | -1.203623 | 1.029363 |
| 9 | C | -2.869856 | 1.280585 | 0.089702 |  | 52 | C | -5.204671 | -0.828620 | 0.932423 |
| 10 | C | -1.522769 | 1.747207 | -0.104651 |  | 53 | C | -4.676993 | -0.417473 | -0.338060 |
| 11 | C | -1.189105 | 3.025229 | 0.384531 |  | 54 | C | -5.511277 | -0.445172 | -1.446165 |
| 12 | C | -2.098516 | 3.807111 | 1.106404 |  | 55 | H | -8.147808 | -1.888183 | 2.348328 |
| 13 | C | -3.390373 | 3.346470 | 1.309798 |  | 56 | H | -7.490693 | -0.798487 | -2.232083 |
| 14 | C | -3.772706 | 2.107958 | 0.784104 |  | 57 | H | -8.440413 | -1.460330 | -0.041926 |
| 15 | H | 0.192912 | -2.959427 | -3.229453 |  | 58 | C | -7.098734 | -1.610234 | 2.284922 |
| 16 | H | -3.746762 | -2.345362 | -1.652455 |  | 59 | C | -4.398690 | -0.914928 | 2.094672 |
| 17 | H | -2.171286 | -3.717721 | -2.960976 |  | 60 | H | -5.110422 | -0.140630 | -2.408796 |
| 18 | H | -1.789913 | 4.777709 | 1.482821 |  | 61 | C | -4.929541 | -1.318959 | 3.296493 |
| 19 | H | -4.114124 | 3.945381 | 1.854157 |  | 62 | C | -6.294683 | -1.663398 | 3.396649 |
| 20 | H | -4.798497 | 1.777715 | 0.904089 |  | 63 | H | -3.346340 | -0.660060 | 2.019136 |
| 21 | C | 0.790386 | 1.413616 | -1.015011 |  | 64 | H | -4.293673 | -1.379114 | 4.174967 |
| 22 | C | 1.579745 | 1.701334 | 0.125896 |  | 65 | H | -6.703588 | -1.979070 | 4.352029 |
| 23 | C | 2.898841 | 2.123471 | 0.035117 |  |  |  |  |  |  |
| 24 | C | 3.422013 | 2.269817 | -1.240381 |  |  |  |  |  |  |
| 25 | C | 2.678857 | 2.003879 | -2.402087 |  |  |  |  |  |  |
| 26 | C | 1.362428 | 1.571115 | -2.289731 |  |  |  |  |  |  |
| 27 | C | 4.793555 | 2.698635 | -2.892556 |  |  |  |  |  |  |
| 28 | C | 5.909626 | 3.056459 | -3.593332 |  |  |  |  |  |  |
| 29 | C | 5.834873 | 3.012380 | -5.008455 |  |  |  |  |  |  |
| 30 | C | 4.618380 | 2.602545 | -5.653411 |  |  |  |  |  |  |
| 31 | C | 3.492327 | 2.241611 | -4.876669 |  |  |  |  |  |  |
| 32 | C | 3.577394 | 2.288667 | -3.503217 |  |  |  |  |  |  |
| 33 | H | 6.822208 | 3.363340 | -3.093066 |  |  |  |  |  |  |
| 34 | H | 2.576715 | 1.933025 | -5.373656 |  |  |  |  |  |  |
| 35 | O | 4.690557 | 2.683740 | -1.524615 |  |  |  |  |  |  |
| 36 | H | 1.137574 | 1.558650 | 1.106956 |  |  |  |  |  |  |
| 37 | H | 0.921554 | -0.849153 | -2.206187 |  |  |  |  |  |  |
| 38 | H | -0.204037 | 3.427414 | 0.176908 |  |  |  |  |  |  |
| 39 | H | 3.497734 | 2.321813 | 0.917182 |  |  |  |  |  |  |
| 40 | H | 0.764926 | 1.376638 | -3.175084 |  |  |  |  |  |  |
| 41 | C | 4.576689 | 2.569542 | -7.072213 |  |  |  |  |  |  |
| 42 | C | 5.671073 | 2.919884 | -7.821544 |  |  |  |  |  |  |
| 43 | C | 6.867916 | 3.322839 | -7.185527 |  |  |  |  |  |  |

**Table S7.** Cartesian coordinates of the optimized T_4_ geometry of **Host 1**.

Energy = ‒1612.06248928 Hartrees

| 1 | C | -0.457833 | -2.639628 | -1.987120 |  | 44 | C | 6.882096 | 3.388706 | -5.853248 |
| --- | --- | --- | --- | --- | --- | --- | --- | --- | --- | --- |
| 2 | C | -0.042123 | -1.339196 | -1.630901 |  | 45 | H | 3.492282 | 2.513917 | -7.536827 |
| 3 | C | -0.986854 | -0.359026 | -1.136120 |  | 46 | H | 5.434988 | 3.155174 | -8.916165 |
| 4 | C | -2.345348 | -0.747833 | -0.981095 |  | 47 | H | 7.605900 | 3.715364 | -7.833305 |
| 5 | C | -2.732558 | -2.092834 | -1.358556 |  | 48 | H | 7.829749 | 3.632987 | -5.380276 |
| 6 | C | -1.786084 | -3.009935 | -1.856438 |  | 49 | C | -6.965223 | -0.300662 | -1.263366 |
| 7 | C | -0.586362 | 0.948256 | -0.788163 |  | 50 | C | -7.434309 | -0.957566 | -0.154064 |
| 8 | C | -3.284734 | 0.168342 | -0.467960 |  | 51 | C | -6.561415 | -1.278318 | 0.915344 |
| 9 | C | -2.881780 | 1.467302 | -0.100444 |  | 52 | C | -5.182781 | -0.911177 | 0.828889 |
| 10 | C | -1.527607 | 1.865382 | -0.275132 |  | 53 | C | -4.715648 | -0.229711 | -0.337002 |
| 11 | C | -1.160437 | 3.224047 | 0.062611 |  | 54 | C | -5.603864 | 0.062375 | -1.351733 |
| 12 | C | -2.106003 | 4.125400 | 0.593353 |  | 55 | H | -8.071759 | -2.230970 | 2.130678 |
| 13 | C | -3.417882 | 3.726374 | 0.792647 |  | 56 | H | -7.637091 | -0.056463 | -2.080948 |
| 14 | C | -3.820639 | 2.419932 | 0.455567 |  | 57 | H | -8.481001 | -1.241167 | -0.079075 |
| 15 | H | 0.276109 | -3.347910 | -2.359431 |  | 58 | C | -7.021831 | -1.955009 | 2.072320 |
| 16 | H | -3.770612 | -2.384712 | -1.249758 |  | 59 | C | -4.323350 | -1.237131 | 1.909239 |
| 17 | H | -2.107442 | -4.010200 | -2.130532 |  | 60 | H | -5.242901 | 0.581176 | -2.235360 |
| 18 | H | -1.795477 | 5.135976 | 0.840873 |  | 61 | C | -4.801809 | -1.894208 | 3.017129 |
| 19 | H | -4.145687 | 4.417757 | 1.206475 |  | 62 | C | -6.163571 | -2.257687 | 3.100910 |
| 20 | H | -4.845769 | 2.106388 | 0.615052 |  | 63 | H | -3.276397 | -0.956821 | 1.846301 |
| 21 | C | 0.833653 | 1.362264 | -0.962130 |  | 64 | H | -4.129683 | -2.136046 | 3.835286 |
| 22 | C | 1.660916 | 1.531543 | 0.163623 |  | 65 | H | -6.529746 | -2.776733 | 3.981895 |
| 23 | C | 2.993807 | 1.919404 | 0.052036 |  |  |  |  |  |  |
| 24 | C | 3.475172 | 2.132705 | -1.227617 |  |  |  |  |  |  |
| 25 | C | 2.684884 | 1.973957 | -2.375263 |  |  |  |  |  |  |
| 26 | C | 1.352702 | 1.585826 | -2.240262 |  |  |  |  |  |  |
| 27 | C | 4.807776 | 2.615234 | -2.898011 |  |  |  |  |  |  |
| 28 | C | 5.911797 | 2.975712 | -3.617612 |  |  |  |  |  |  |
| 29 | C | 5.786560 | 3.024246 | -5.028397 |  |  |  |  |  |  |
| 30 | C | 4.533463 | 2.701202 | -5.652521 |  |  |  |  |  |  |
| 31 | C | 3.421864 | 2.334454 | -4.858665 |  |  |  |  |  |  |
| 32 | C | 3.556092 | 2.291217 | -3.488820 |  |  |  |  |  |  |
| 33 | H | 6.852450 | 3.217131 | -3.133657 |  |  |  |  |  |  |
| 34 | H | 2.478033 | 2.092217 | -5.339475 |  |  |  |  |  |  |
| 35 | O | 4.752682 | 2.517310 | -1.532203 |  |  |  |  |  |  |
| 36 | H | 1.242046 | 1.349825 | 1.148983 |  |  |  |  |  |  |
| 37 | H | 1.002540 | -1.063240 | -1.710916 |  |  |  |  |  |  |
| 38 | H | -0.140917 | 3.547727 | -0.110226 |  |  |  |  |  |  |
| 39 | H | 3.627885 | 2.046089 | 0.922610 |  |  |  |  |  |  |
| 40 | H | 0.717331 | 1.459885 | -3.112139 |  |  |  |  |  |  |
| 41 | C | 4.442207 | 2.759302 | -7.068632 |  |  |  |  |  |  |
| 42 | C | 5.522572 | 3.115808 | -7.834496 |  |  |  |  |  |  |
| 43 | C | 6.755642 | 3.433975 | -7.218921 |  |  |  |  |  |  |

**Table S8.** Cartesian coordinates of the optimized S_0_ geometry of **Host 2**.

Energy = ‒1689.49033550 Hartrees

| 1 | C | -1.078024 | 0.674870 | 3.892720 |  | 44 | C | -5.628010 | 3.574427 | -0.117057 |
| --- | --- | --- | --- | --- | --- | --- | --- | --- | --- | --- |
| 2 | C | -0.416888 | 0.230104 | 2.782744 |  | 45 | H | -7.606113 | 0.864665 | -0.593584 |
| 3 | C | -1.021534 | 0.273321 | 1.488865 |  | 46 | H | -7.737576 | 3.329981 | -0.487407 |
| 4 | C | -2.346139 | 0.826601 | 1.370841 |  | 47 | H | -5.670846 | 4.658159 | -0.061125 |
| 5 | C | -3.004003 | 1.268578 | 2.560281 |  | 48 | H | -3.506568 | 3.489521 | 0.220644 |
| 6 | C | -2.394104 | 1.193536 | 3.781099 |  | 49 | C | -5.489996 | -0.714264 | -0.340376 |
| 7 | C | -0.352259 | -0.201064 | 0.344750 |  | 50 | C | -6.117775 | -1.349188 | -1.419495 |
| 8 | C | -2.969382 | 0.902309 | 0.110128 |  | 51 | C | -4.912620 | -1.508647 | 0.657700 |
| 9 | C | -2.292485 | 0.443366 | -1.036177 |  | 52 | C | -6.161521 | -2.737170 | -1.504326 |
| 10 | C | -0.974091 | -0.124284 | -0.916022 |  | 53 | H | -6.560600 | -0.744423 | -2.206552 |
| 11 | C | -0.331251 | -0.604373 | -2.098346 |  | 54 | C | -4.959448 | -2.896306 | 0.574366 |
| 12 | C | -0.931899 | -0.510308 | -3.321666 |  | 55 | H | -4.440067 | -1.035739 | 1.512789 |
| 13 | C | -2.219128 | 0.074418 | -3.443639 |  | 56 | C | -5.581299 | -3.516010 | -0.506938 |
| 14 | C | -2.875340 | 0.533342 | -2.336899 |  | 57 | H | -6.645647 | -3.210167 | -2.354035 |
| 15 | H | -0.597026 | 0.631192 | 4.865532 |  | 58 | H | -4.512300 | -3.496033 | 1.362086 |
| 16 | H | -4.008370 | 1.670799 | 2.477152 |  | 59 | H | -5.614205 | -4.599796 | -0.571009 |
| 17 | H | -2.915889 | 1.534713 | 4.670500 |  | 60 | H | 2.556086 | -3.700427 | 1.434452 |
| 18 | H | -0.423577 | -0.882664 | -4.206357 |  | 61 | H | 2.015448 | 0.991124 | -0.235535 |
| 19 | H | -2.683217 | 0.154543 | -4.422334 |  | 62 | C | 7.401098 | 2.263818 | -1.011799 |
| 20 | H | -3.858866 | 0.980532 | -2.433369 |  | 63 | C | 8.767138 | 2.328186 | -1.114443 |
| 21 | C | 1.014947 | -0.782403 | 0.469957 |  | 64 | C | 9.559687 | 1.212434 | -0.756990 |
| 22 | C | 1.178708 | -2.098763 | 0.938692 |  | 65 | C | 8.970648 | 0.058422 | -0.305197 |
| 23 | C | 2.434418 | -2.684598 | 1.074904 |  | 66 | H | 6.787350 | 3.118425 | -1.285198 |
| 24 | C | 3.525273 | -1.907611 | 0.726498 |  | 67 | H | 9.245060 | 3.235946 | -1.470523 |
| 25 | C | 3.404676 | -0.592323 | 0.255509 |  | 68 | H | 10.640814 | 1.272394 | -0.841554 |
| 26 | C | 2.137783 | -0.025747 | 0.125771 |  | 69 | H | 9.579609 | -0.798924 | -0.030429 |
| 27 | C | 5.583484 | -1.249667 | 0.366133 |  |  |  |  |  |  |
| 28 | C | 6.947044 | -1.234430 | 0.280888 |  |  |  |  |  |  |
| 29 | C | 7.560477 | -0.045269 | -0.185608 |  |  |  |  |  |  |
| 30 | C | 6.754521 | 1.087259 | -0.548528 |  |  |  |  |  |  |
| 31 | C | 5.346007 | 1.016463 | -0.440867 |  |  |  |  |  |  |
| 32 | C | 4.761838 | -0.144592 | 0.013625 |  |  |  |  |  |  |
| 33 | H | 7.544162 | -2.096819 | 0.558547 |  |  |  |  |  |  |
| 34 | H | 4.744923 | 1.878371 | -0.717514 |  |  |  |  |  |  |
| 35 | O | 4.830996 | -2.311447 | 0.795546 |  |  |  |  |  |  |
| 36 | H | 0.294010 | -2.672244 | 1.198877 |  |  |  |  |  |  |
| 37 | H | 0.588926 | -0.166870 | 2.870456 |  |  |  |  |  |  |
| 38 | H | 0.653468 | -1.050190 | -2.007253 |  |  |  |  |  |  |
| 39 | C | -4.414008 | 2.917471 | 0.047504 |  |  |  |  |  |  |
| 40 | C | -4.321034 | 1.522084 | -0.020081 |  |  |  |  |  |  |
| 41 | C | -5.490152 | 0.768160 | -0.255320 |  |  |  |  |  |  |
| 42 | C | -6.704453 | 1.447400 | -0.426252 |  |  |  |  |  |  |
| 43 | C | -6.780398 | 2.833035 | -0.358886 |  |  |  |  |  |  |

**Table S9.** Cartesian coordinates of the optimized S_1_ geometry of **Host 2**.

Energy = ‒1689.38561656 Hartrees

| 1 | C | 0.273256 | -3.576170 | 0.091784 |  | 44 | C | 5.659325 | -1.494933 | -3.226410 |
| --- | --- | --- | --- | --- | --- | --- | --- | --- | --- | --- |
| 2 | C | -0.108467 | -2.240359 | 0.275198 |  | 45 | H | 7.658809 | -0.598976 | -0.639756 |
| 3 | C | 0.733915 | -1.165962 | -0.065800 |  | 46 | H | 7.799060 | -1.435000 | -2.961447 |
| 4 | C | 2.053854 | -1.473687 | -0.552559 |  | 47 | H | 5.705743 | -1.873488 | -4.243391 |
| 5 | C | 2.421463 | -2.827136 | -0.691142 |  | 48 | H | 3.512253 | -1.421056 | -3.193461 |
| 6 | C | 1.536221 | -3.870747 | -0.396229 |  | 49 | C | 5.497701 | -0.041859 | 0.809214 |
| 7 | C | 0.355545 | 0.203888 | 0.132209 |  | 50 | C | 6.334133 | 1.004168 | 1.217253 |
| 8 | C | 2.983517 | -0.418188 | -0.798538 |  | 51 | C | 4.678383 | -0.656682 | 1.764981 |
| 9 | C | 2.605832 | 0.946871 | -0.613613 |  | 52 | C | 6.347751 | 1.430590 | 2.541871 |
| 10 | C | 1.277685 | 1.262144 | -0.160695 |  | 53 | H | 6.964896 | 1.498261 | 0.482824 |
| 11 | C | 0.903573 | 2.618043 | -0.104419 |  | 54 | C | 4.694219 | -0.231398 | 3.089468 |
| 12 | C | 1.802129 | 3.651525 | -0.398689 |  | 55 | H | 4.044103 | -1.487464 | 1.471211 |
| 13 | C | 3.089829 | 3.345355 | -0.809372 |  | 56 | C | 5.526214 | 0.814537 | 3.482292 |
| 14 | C | 3.474441 | 2.005964 | -0.935885 |  | 57 | H | 6.996610 | 2.250129 | 2.837645 |
| 15 | H | -0.420173 | -4.370336 | 0.351604 |  | 58 | H | 4.057889 | -0.724584 | 3.818781 |
| 16 | H | 3.426191 | -3.063158 | -1.024555 |  | 59 | H | 5.535521 | 1.146392 | 4.516540 |
| 17 | H | 1.853695 | -4.900140 | -0.531746 |  | 60 | H | -2.462866 | 2.232997 | 3.247562 |
| 18 | H | 1.475043 | 4.683917 | -0.320807 |  | 61 | H | -2.049394 | -0.517654 | -0.920381 |
| 19 | H | 3.796344 | 4.132686 | -1.053770 |  | 62 | C | -7.468052 | -1.300847 | -1.998087 |
| 20 | H | 4.465261 | 1.773197 | -1.309683 |  | 63 | C | -8.838387 | -1.345558 | -2.043590 |
| 21 | C | -0.983914 | 0.531125 | 0.639300 |  | 64 | C | -9.603591 | -0.694420 | -1.048699 |
| 22 | C | -1.127795 | 1.319138 | 1.808096 |  | 65 | C | -8.984059 | -0.013110 | -0.030708 |
| 23 | C | -2.367160 | 1.645100 | 2.341323 |  | 66 | H | -6.875066 | -1.799665 | -2.760449 |
| 24 | C | -3.482141 | 1.173718 | 1.665615 |  | 67 | H | -9.340043 | -1.881213 | -2.843937 |
| 25 | C | -3.392132 | 0.395042 | 0.500101 |  | 68 | H | -10.687980 | -0.735102 | -1.092620 |
| 26 | C | -2.141376 | 0.068881 | -0.011481 |  | 69 | H | -9.572470 | 0.487378 | 0.733862 |
| 27 | C | -5.558795 | 0.772963 | 1.098177 |  |  |  |  |  |  |
| 28 | C | -6.924261 | 0.757373 | 1.097807 |  |  |  |  |  |  |
| 29 | C | -7.569569 | 0.054833 | 0.048912 |  |  |  |  |  |  |
| 30 | C | -6.791023 | -0.606507 | -0.961135 |  |  |  |  |  |  |
| 31 | C | -5.378002 | -0.558129 | -0.910403 |  |  |  |  |  |  |
| 32 | C | -4.763117 | 0.127354 | 0.113237 |  |  |  |  |  |  |
| 33 | H | -7.499758 | 1.260926 | 1.867557 |  |  |  |  |  |  |
| 34 | H | -4.798112 | -1.061180 | -1.679392 |  |  |  |  |  |  |
| 35 | O | -4.776054 | 1.402294 | 2.032767 |  |  |  |  |  |  |
| 36 | H | -0.229127 | 1.654883 | 2.316054 |  |  |  |  |  |  |
| 37 | H | -1.078250 | -2.031892 | 0.712220 |  |  |  |  |  |  |
| 38 | H | -0.118214 | 2.872563 | 0.153388 |  |  |  |  |  |  |
| 39 | C | 4.427696 | -1.248575 | -2.634336 |  |  |  |  |  |  |
| 40 | C | 4.327843 | -0.740656 | -1.329147 |  |  |  |  |  |  |
| 41 | C | 5.516145 | -0.510062 | -0.597020 |  |  |  |  |  |  |
| 42 | C | 6.749396 | -0.760513 | -1.212130 |  |  |  |  |  |  |
| 43 | C | 6.828834 | -1.243329 | -2.512549 |  |  |  |  |  |  |

**Table S10.** Cartesian coordinates of the optimized T_3_ geometry of **Host 2**.

Energy = ‒1689.38245149 Hartrees

| 1 | C | -0.638181 | -3.199853 | 0.781455 |  | 44 | C | 6.050867 | -2.830690 | 0.695507 |
| --- | --- | --- | --- | --- | --- | --- | --- | --- | --- | --- |
| 2 | C | -0.684287 | -1.947831 | 0.136741 |  | 45 | H | 6.506597 | -0.958283 | 3.483160 |
| 3 | C | 0.485548 | -1.098632 | 0.064061 |  | 46 | H | 7.631289 | -2.714353 | 2.156797 |
| 4 | C | 1.707303 | -1.577168 | 0.613433 |  | 47 | H | 6.529582 | -3.607513 | 0.106559 |
| 5 | C | 1.708008 | -2.860893 | 1.283227 |  | 48 | H | 4.335073 | -2.698933 | -0.593795 |
| 6 | C | 0.541244 | -3.647244 | 1.354806 |  | 49 | C | 4.175366 | 0.236390 | 3.047195 |
| 7 | C | 0.440490 | 0.170371 | -0.548662 |  | 50 | C | 4.931670 | 1.351507 | 3.429886 |
| 8 | C | 2.875546 | -0.793358 | 0.521667 |  | 51 | C | 2.858624 | 0.131141 | 3.511974 |
| 9 | C | 2.834479 | 0.465817 | -0.111702 |  | 52 | C | 4.387443 | 2.336639 | 4.247815 |
| 10 | C | 1.608238 | 0.956984 | -0.634353 |  | 53 | H | 5.951451 | 1.450953 | 3.067307 |
| 11 | C | 1.590761 | 2.278294 | -1.231556 |  | 54 | C | 2.316006 | 1.114046 | 4.332809 |
| 12 | C | 2.768943 | 3.045543 | -1.342108 |  | 55 | H | 2.263532 | -0.735898 | 3.242299 |
| 13 | C | 3.971914 | 2.548566 | -0.870339 |  | 56 | C | 3.076510 | 2.221156 | 4.701909 |
| 14 | C | 4.025914 | 1.278194 | -0.261350 |  | 57 | H | 4.987698 | 3.198135 | 4.526382 |
| 15 | H | -1.535418 | -3.809956 | 0.823165 |  | 58 | H | 1.295074 | 1.011543 | 4.689552 |
| 16 | H | 2.627810 | -3.211372 | 1.737064 |  | 59 | H | 2.649471 | 2.989220 | 5.340259 |
| 17 | H | 0.576966 | -4.607398 | 1.860605 |  | 60 | H | -3.869752 | 1.916664 | -0.040015 |
| 18 | H | 2.721269 | 4.029289 | -1.799161 |  | 61 | H | -0.249807 | 0.418236 | -3.161794 |
| 19 | H | 4.882328 | 3.133030 | -0.962253 |  | 62 | C | -2.825871 | 1.408385 | -8.020253 |
| 20 | H | 4.971117 | 0.886692 | 0.095190 |  | 63 | C | -3.660019 | 1.734024 | -9.059024 |
| 21 | C | -0.842768 | 0.680638 | -1.106250 |  | 64 | C | -4.947963 | 2.257448 | -8.798567 |
| 22 | C | -1.867745 | 1.102864 | -0.239450 |  | 65 | C | -5.373664 | 2.443767 | -7.507374 |
| 23 | C | -3.084165 | 1.589276 | -0.712085 |  | 66 | H | -1.835071 | 1.005698 | -8.215036 |
| 24 | C | -3.242851 | 1.640370 | -2.085695 |  | 67 | H | -3.334459 | 1.591142 | -10.085104 |
| 25 | C | -2.246919 | 1.229664 | -2.983713 |  | 68 | H | -5.601839 | 2.512531 | -9.627404 |
| 26 | C | -1.036855 | 0.745543 | -2.488662 |  | 69 | H | -6.363411 | 2.845878 | -7.307082 |
| 27 | C | -4.107264 | 1.969093 | -4.071576 |  |  |  |  |  |  |
| 28 | C | -4.973525 | 2.308806 | -5.071960 |  |  |  |  |  |  |
| 29 | C | -4.538657 | 2.118491 | -6.407254 |  |  |  |  |  |  |
| 30 | C | -3.230253 | 1.586652 | -6.670527 |  |  |  |  |  |  |
| 31 | C | -2.373542 | 1.252079 | -5.596045 |  |  |  |  |  |  |
| 32 | C | -2.807704 | 1.441401 | -4.302923 |  |  |  |  |  |  |
| 33 | H | -5.959113 | 2.709778 | -4.859436 |  |  |  |  |  |  |
| 34 | H | -1.385769 | 0.850343 | -5.804919 |  |  |  |  |  |  |
| 35 | O | -4.364235 | 2.086907 | -2.730676 |  |  |  |  |  |  |
| 36 | H | -1.697323 | 1.049687 | 0.831744 |  |  |  |  |  |  |
| 37 | H | -1.603799 | -1.609755 | -0.326188 |  |  |  |  |  |  |
| 38 | H | 0.647543 | 2.679107 | -1.582804 |  |  |  |  |  |  |
| 39 | C | 4.815500 | -2.325135 | 0.306308 |  |  |  |  |  |  |
| 40 | C | 4.170498 | -1.322069 | 1.040030 |  |  |  |  |  |  |
| 41 | C | 4.789339 | -0.820501 | 2.204584 |  |  |  |  |  |  |
| 42 | C | 6.038240 | -1.335706 | 2.578259 |  |  |  |  |  |  |
| 43 | C | 6.667290 | -2.328963 | 1.837637 |  |  |  |  |  |  |

**Table S11.** Cartesian coordinates of the optimized S_0_ geometry of **MADN**.

Energy = ‒1346.69536634 Hartrees

| 1 | C | 0.600003 | -3.926039 | -0.122754 |  | 44 | C | -2.950871 | -0.232232 | 0.345529 |
| --- | --- | --- | --- | --- | --- | --- | --- | --- | --- | --- |
| 2 | C | 1.301435 | -2.753861 | -0.166731 |  | 45 | H | -7.585125 | -0.495506 | 1.854153 |
| 3 | C | 0.641410 | -1.488988 | -0.094708 |  | 46 | H | -2.855512 | -0.901941 | 2.401959 |
| 4 | C | -0.789917 | -1.473643 | 0.057997 |  | 47 | H | -5.298586 | -0.890946 | 2.740421 |
| 5 | C | -1.483581 | -2.721954 | 0.081928 |  | 48 | C | -7.163782 | -0.206099 | 0.894712 |
| 6 | C | -0.813638 | -3.909969 | -0.006278 |  | 49 | C | -6.069675 | 0.537964 | -1.573161 |
| 7 | C | 1.357817 | -0.276971 | -0.158061 |  | 50 | H | -3.368325 | 0.430313 | -1.642880 |
| 8 | C | -1.472613 | -0.245304 | 0.165803 |  | 51 | C | -7.430487 | 0.543245 | -1.387990 |
| 9 | C | -0.754359 | 0.963604 | 0.114398 |  | 52 | C | -7.983086 | 0.167986 | -0.142274 |
| 10 | C | 0.674723 | 0.950420 | -0.062654 |  | 53 | H | -5.641728 | 0.825976 | -2.530060 |
| 11 | C | 1.361229 | 2.201045 | -0.148468 |  | 54 | H | -8.088868 | 0.836553 | -2.200486 |
| 12 | C | 0.713864 | 3.402184 | -0.037227 |  | 55 | H | -9.060916 | 0.176654 | -0.008774 |
| 13 | C | -0.696524 | 3.398829 | 0.177320 |  | 56 | C | 1.445974 | 4.708531 | -0.131803 |
| 14 | C | -1.399366 | 2.232282 | 0.247771 |  | 57 | H | 1.316218 | 5.300171 | 0.781721 |
| 15 | H | 1.125120 | -4.875194 | -0.178704 |  | 58 | H | 2.516736 | 4.557576 | -0.288685 |
| 16 | H | -2.564938 | -2.712668 | 0.169440 |  | 59 | H | 1.063565 | 5.315775 | -0.960298 |
| 17 | H | -1.363481 | -4.846436 | 0.013449 |  |  |  |  |  |  |
| 18 | H | -1.215770 | 4.348352 | 0.284233 |  |  |  |  |  |  |
| 19 | H | -2.471748 | 2.253986 | 0.409438 |  |  |  |  |  |  |
| 20 | H | 2.382584 | -2.770150 | -0.255622 |  |  |  |  |  |  |
| 21 | H | 2.434901 | 2.186275 | -0.307847 |  |  |  |  |  |  |
| 22 | C | 7.338679 | 0.229185 | 1.426760 |  |  |  |  |  |  |
| 23 | C | 5.978427 | 0.257568 | 1.613927 |  |  |  |  |  |  |
| 24 | C | 5.093973 | -0.034368 | 0.544896 |  |  |  |  |  |  |
| 25 | C | 5.639795 | -0.360548 | -0.732850 |  |  |  |  |  |  |
| 26 | C | 7.047569 | -0.380949 | -0.894079 |  |  |  |  |  |  |
| 27 | C | 7.878696 | -0.092815 | 0.160766 |  |  |  |  |  |  |
| 28 | H | 3.274643 | 0.234524 | 1.682407 |  |  |  |  |  |  |
| 29 | H | 8.006315 | 0.454559 | 2.253279 |  |  |  |  |  |  |
| 30 | H | 5.560202 | 0.504606 | 2.586449 |  |  |  |  |  |  |
| 31 | C | 3.686675 | -0.013209 | 0.707075 |  |  |  |  |  |  |
| 32 | C | 4.749722 | -0.653136 | -1.796182 |  |  |  |  |  |  |
| 33 | H | 7.459221 | -0.629397 | -1.869111 |  |  |  |  |  |  |
| 34 | H | 8.956234 | -0.111714 | 0.025925 |  |  |  |  |  |  |
| 35 | C | 3.391016 | -0.625572 | -1.606673 |  |  |  |  |  |  |
| 36 | C | 2.836029 | -0.300966 | -0.339266 |  |  |  |  |  |  |
| 37 | H | 5.160954 | -0.899497 | -2.771958 |  |  |  |  |  |  |
| 38 | H | 2.718708 | -0.849991 | -2.429873 |  |  |  |  |  |  |
| 39 | C | -3.518526 | -0.609893 | 1.592569 |  |  |  |  |  |  |
| 40 | C | -4.877718 | -0.603650 | 1.780066 |  |  |  |  |  |  |
| 41 | C | -5.755844 | -0.222545 | 0.734943 |  |  |  |  |  |  |
| 42 | C | -5.197317 | 0.157505 | -0.522216 |  |  |  |  |  |  |
| 43 | C | -3.789800 | 0.142049 | -0.682844 |  |  |  |  |  |  |

**Table S12.** Cartesian coordinates of the optimized S_1_ geometry of **MADN**.

Energy = ‒1346.59317872 Hartrees

| 1 | C | 0.570947 | -4.076116 | -0.008237 |  | 44 | C | -2.951451 | -0.340016 | 0.269147 |
| --- | --- | --- | --- | --- | --- | --- | --- | --- | --- | --- |
| 2 | C | 1.282718 | -2.874783 | 0.033828 |  | 45 | H | -7.646110 | -0.896076 | 1.531805 |
| 3 | C | 0.639109 | -1.623103 | -0.036048 |  | 46 | H | -2.961294 | -1.695986 | 1.962384 |
| 4 | C | -0.799159 | -1.599961 | 0.004927 |  | 47 | H | -5.399008 | -1.676804 | 2.257412 |
| 5 | C | -1.484275 | -2.825246 | -0.111010 |  | 48 | C | -7.186578 | -0.307612 | 0.741515 |
| 6 | C | -0.812908 | -4.050596 | -0.116560 |  | 49 | C | -5.997233 | 1.204249 | -1.293298 |
| 7 | C | 1.372835 | -0.394596 | -0.113462 |  | 50 | H | -3.310597 | 1.001065 | -1.366171 |
| 8 | C | -1.491574 | -0.350882 | 0.129515 |  | 51 | C | -7.362513 | 1.204813 | -1.136958 |
| 9 | C | -0.753350 | 0.875170 | 0.124001 |  | 52 | C | -7.964354 | 0.444847 | -0.110277 |
| 10 | C | 0.673630 | 0.856979 | -0.060649 |  | 53 | H | -5.531933 | 1.789583 | -2.082394 |
| 11 | C | 1.332717 | 2.087826 | -0.224740 |  | 54 | H | -7.985378 | 1.792353 | -1.805322 |
| 12 | C | 0.690171 | 3.329359 | -0.081301 |  | 55 | H | -9.044307 | 0.454743 | 0.004020 |
| 13 | C | -0.666698 | 3.327904 | 0.226414 |  | 56 | C | 1.464060 | 4.601821 | -0.249585 |
| 14 | C | -1.367287 | 2.125124 | 0.329513 |  | 57 | H | 0.826783 | 5.478613 | -0.111858 |
| 15 | H | 1.106489 | -5.019622 | 0.034308 |  | 58 | H | 2.288268 | 4.664741 | 0.470296 |
| 16 | H | -2.564953 | -2.817244 | -0.197265 |  | 59 | H | 1.911848 | 4.660940 | -1.248413 |
| 17 | H | -1.380051 | -4.973189 | -0.194443 |  |  |  |  |  |  |
| 18 | H | -1.190745 | 4.267029 | 0.381452 |  |  |  |  |  |  |
| 19 | H | -2.422655 | 2.155625 | 0.576051 |  |  |  |  |  |  |
| 20 | H | 2.362779 | -2.906387 | 0.122243 |  |  |  |  |  |  |
| 21 | H | 2.388929 | 2.082998 | -0.474185 |  |  |  |  |  |  |
| 22 | C | 7.291371 | 0.929490 | 1.192769 |  |  |  |  |  |  |
| 23 | C | 5.926806 | 0.968194 | 1.350941 |  |  |  |  |  |  |
| 24 | C | 5.073072 | 0.260000 | 0.464546 |  |  |  |  |  |  |
| 25 | C | 5.660183 | -0.501594 | -0.592705 |  |  |  |  |  |  |
| 26 | C | 7.065582 | -0.517993 | -0.731156 |  |  |  |  |  |  |
| 27 | C | 7.867794 | 0.182616 | 0.142332 |  |  |  |  |  |  |
| 28 | H | 3.235580 | 0.849042 | 1.421822 |  |  |  |  |  |  |
| 29 | H | 7.933224 | 1.475453 | 1.878209 |  |  |  |  |  |  |
| 30 | H | 5.481035 | 1.543301 | 2.158640 |  |  |  |  |  |  |
| 31 | C | 3.668425 | 0.283289 | 0.600794 |  |  |  |  |  |  |
| 32 | C | 4.797012 | -1.215565 | -1.466922 |  |  |  |  |  |  |
| 33 | H | 7.505672 | -1.096325 | -1.539756 |  |  |  |  |  |  |
| 34 | H | 8.947447 | 0.160863 | 0.026903 |  |  |  |  |  |  |
| 35 | C | 3.439044 | -1.190626 | -1.299235 |  |  |  |  |  |  |
| 36 | C | 2.831144 | -0.426831 | -0.255165 |  |  |  |  |  |  |
| 37 | H | 5.233770 | -1.780462 | -2.286864 |  |  |  |  |  |  |
| 38 | H | 2.797095 | -1.728554 | -1.990232 |  |  |  |  |  |  |
| 39 | C | -3.584541 | -1.115290 | 1.289093 |  |  |  |  |  |  |
| 40 | C | -4.942916 | -1.100965 | 1.455823 |  |  |  |  |  |  |
| 41 | C | -5.780933 | -0.332530 | 0.604004 |  |  |  |  |  |  |
| 42 | C | -5.168182 | 0.441750 | -0.429028 |  |  |  |  |  |  |
| 43 | C | -3.762985 | 0.423258 | -0.564266 |  |  |  |  |  |  |

**Table S13.** Cartesian coordinates of the optimized T_4_ geometry of **MADN**.

Energy = ‒1346.58760332 Hartrees

| 1 | C | 0.569998 | -3.999911 | -0.101419 |  | 44 | C | -2.940499 | -0.245082 | 0.341910 |
| --- | --- | --- | --- | --- | --- | --- | --- | --- | --- | --- |
| 2 | C | 1.298715 | -2.792451 | -0.140246 |  | 45 | H | -7.580736 | -0.526618 | 1.830658 |
| 3 | C | 0.630219 | -1.512120 | -0.090211 |  | 46 | H | -2.855535 | -0.992213 | 2.371650 |
| 4 | C | -0.785723 | -1.491232 | 0.057039 |  | 47 | H | -5.299841 | -0.975468 | 2.705075 |
| 5 | C | -1.498787 | -2.752823 | 0.067866 |  | 48 | C | -7.154979 | -0.204574 | 0.883633 |
| 6 | C | -0.812189 | -3.978881 | -0.007423 |  | 49 | C | -6.049248 | 0.623038 | -1.552118 |
| 7 | C | 1.347137 | -0.300551 | -0.159089 |  | 50 | H | -3.348505 | 0.495005 | -1.621237 |
| 8 | C | -1.462945 | -0.261245 | 0.165725 |  | 51 | C | -7.410737 | 0.632706 | -1.369413 |
| 9 | C | -0.741981 | 0.952050 | 0.113654 |  | 52 | C | -7.969135 | 0.215625 | -0.139980 |
| 10 | C | 0.666164 | 0.933517 | -0.068053 |  | 53 | H | -5.616970 | 0.943588 | -2.496639 |
| 11 | C | 1.364631 | 2.195489 | -0.175725 |  | 54 | H | -8.064737 | 0.962168 | -2.171530 |
| 12 | C | 0.693189 | 3.441374 | -0.050808 |  | 55 | H | -9.047114 | 0.228491 | -0.008070 |
| 13 | C | -0.677739 | 3.436378 | 0.173928 |  | 56 | C | 1.474963 | 4.714660 | -0.162867 |
| 14 | C | -1.398191 | 2.236921 | 0.259850 |  | 57 | H | 0.832819 | 5.591190 | -0.045791 |
| 15 | H | 1.103774 | -4.944384 | -0.146487 |  | 58 | H | 2.262067 | 4.765270 | 0.599023 |
| 16 | H | -2.581116 | -2.739417 | 0.120289 |  | 59 | H | 1.974644 | 4.787070 | -1.136444 |
| 17 | H | -1.376166 | -4.906625 | 0.008369 |  |  |  |  |  |  |
| 18 | H | -1.206279 | 4.379675 | 0.285957 |  |  |  |  |  |  |
| 19 | H | -2.464354 | 2.258180 | 0.451181 |  |  |  |  |  |  |
| 20 | H | 2.380820 | -2.815459 | -0.193930 |  |  |  |  |  |  |
| 21 | H | 2.431239 | 2.183824 | -0.371268 |  |  |  |  |  |  |
| 22 | C | 7.318931 | 0.346299 | 1.406730 |  |  |  |  |  |  |
| 23 | C | 5.957502 | 0.377419 | 1.587783 |  |  |  |  |  |  |
| 24 | C | 5.078539 | 0.011505 | 0.537489 |  |  |  |  |  |  |
| 25 | C | 5.630855 | -0.393034 | -0.714996 |  |  |  |  |  |  |
| 26 | C | 7.039123 | -0.413663 | -0.870717 |  |  |  |  |  |  |
| 27 | C | 7.865192 | -0.052433 | 0.165842 |  |  |  |  |  |  |
| 28 | H | 3.253583 | 0.338368 | 1.650748 |  |  |  |  |  |  |
| 29 | H | 7.982193 | 0.628902 | 2.219061 |  |  |  |  |  |  |
| 30 | H | 5.534619 | 0.683665 | 2.541285 |  |  |  |  |  |  |
| 31 | C | 3.670417 | 0.032780 | 0.694018 |  |  |  |  |  |  |
| 32 | C | 4.746210 | -0.760808 | -1.759345 |  |  |  |  |  |  |
| 33 | H | 7.455597 | -0.721459 | -1.826572 |  |  |  |  |  |  |
| 34 | H | 8.943233 | -0.072423 | 0.035344 |  |  |  |  |  |  |
| 35 | C | 3.386259 | -0.730910 | -1.575638 |  |  |  |  |  |  |
| 36 | C | 2.824377 | -0.328129 | -0.333984 |  |  |  |  |  |  |
| 37 | H | 5.162276 | -1.066353 | -2.716177 |  |  |  |  |  |  |
| 38 | H | 2.718241 | -1.011286 | -2.385095 |  |  |  |  |  |  |
| 39 | C | -3.514386 | -0.665737 | 1.572012 |  |  |  |  |  |  |
| 40 | C | -4.874425 | -0.655544 | 1.757109 |  |  |  |  |  |  |
| 41 | C | -5.746943 | -0.226968 | 0.725877 |  |  |  |  |  |  |
| 42 | C | -5.182355 | 0.196092 | -0.514935 |  |  |  |  |  |  |
| 43 | C | -3.774449 | 0.174631 | -0.673395 |  |  |  |  |  |  |

## 3. Photophysical properties


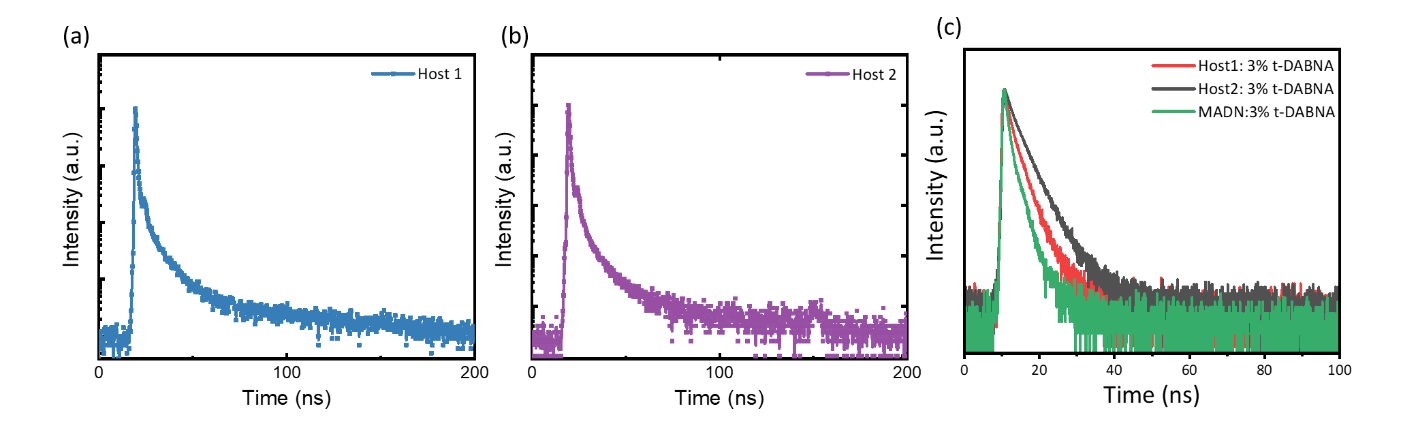


**Figure S17.** transient-PL decay properties of films of (a) **Host 1**, (b) **Host 2**; (3) **Host 1, Host 2** and MADN, respectively, doped with 3 wt% t-DABNA (excited at 365 nm).


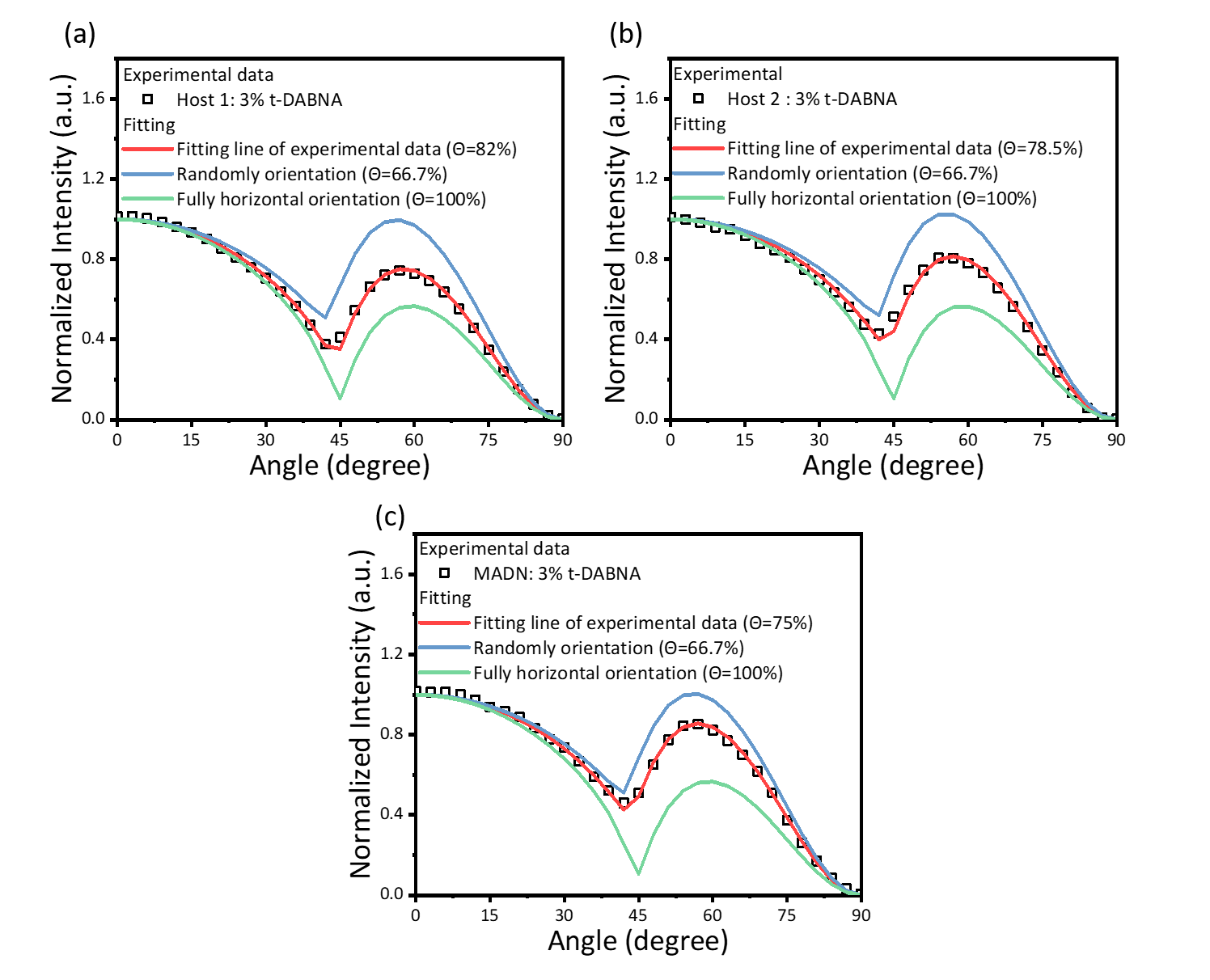


**Figure S18.** Measured results of angle dependent PL (ADPL) intensity of p-polarized light from the emitter t-DABNA doped in host (a) **Host 1**, (b) **Host 2** and (c) MADN films. (Solid lines: fitted line).

**Figure S19**. Cyclic voltammograms of all complexes in degassed CH_2_Cl_2_ for oxidation

**Table S14.** Electrochemical^a^ data of the investigated host materials.

|  | *E*_ox_*^b^*(V) vs. Fc^+/0^ | *E*_HOMO_*^c^*  (eV) | *E*_g_*^d^*  (eV) | *E*_LUMO_*^e^*  (eV) |
| --- | --- | --- | --- | --- |
| **Host 1** | 0.76 | -5.59 | 3.01 | -2.58 |
| **Host 2** | 0.79 | -5.56 | 2.99 | -2.57 |
| MADN | 0.71 | -5.51 | 2.95 | -2.55 |

*^a^* In CH_2_Cl_2_ solution with 0.1 M *^n^*Bu_4_NPF_6_ as supporting electrolyte at 298 K and values are recorded vs. SCE; scan rate: 100 mV s^-1^.

*^b^* The values are recorded *versus* Fc^+/0^.

*^c^* *E*_HOMO_ levels were calculated from electrochemical potentials, *E*_HOMO_= −e(*E*_ox_+4.8).

*^d^ E*_g_= 1240/λ_onset_ (UV).

*^f^* LUMO = HOMO + *E_g_*


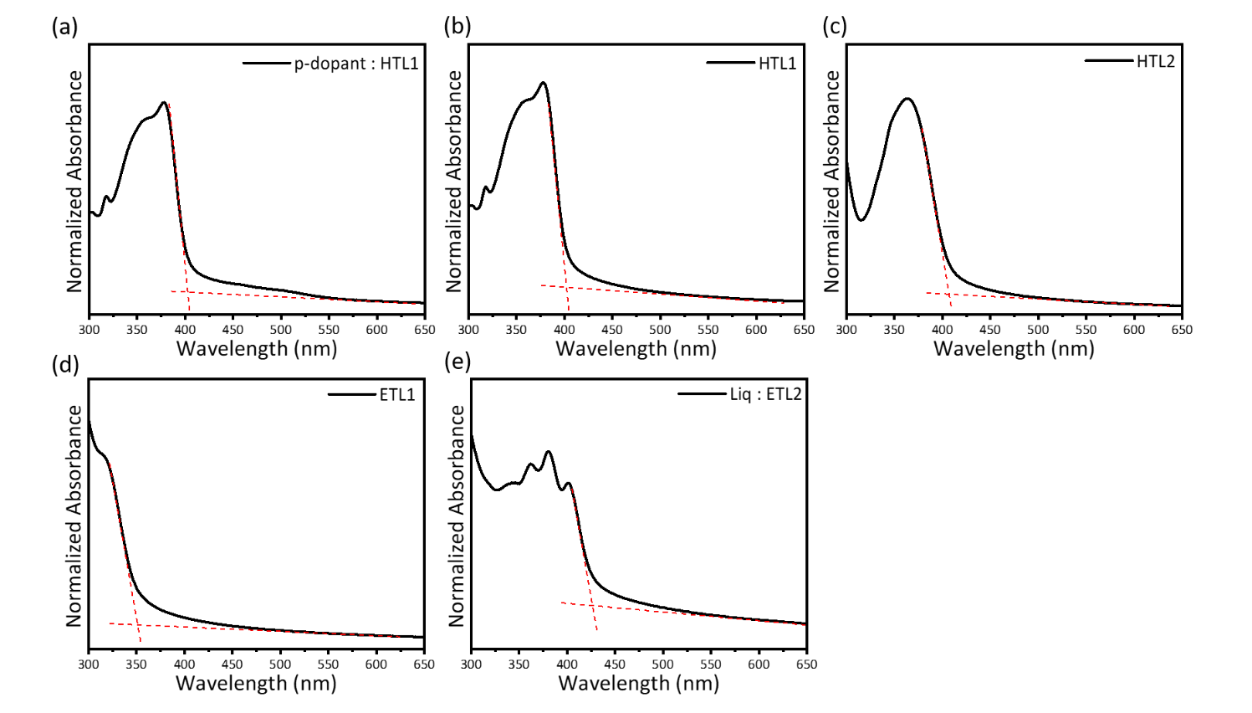


**Figure S20**. UV-vis spectra of organic layers in TTU device: (a) 3 wt% p-dopant: HTL1, (b)HTL1, (c) HTL2, (d) ETL1 and (e) 50 wt% Liq : ETL2 in film samples.


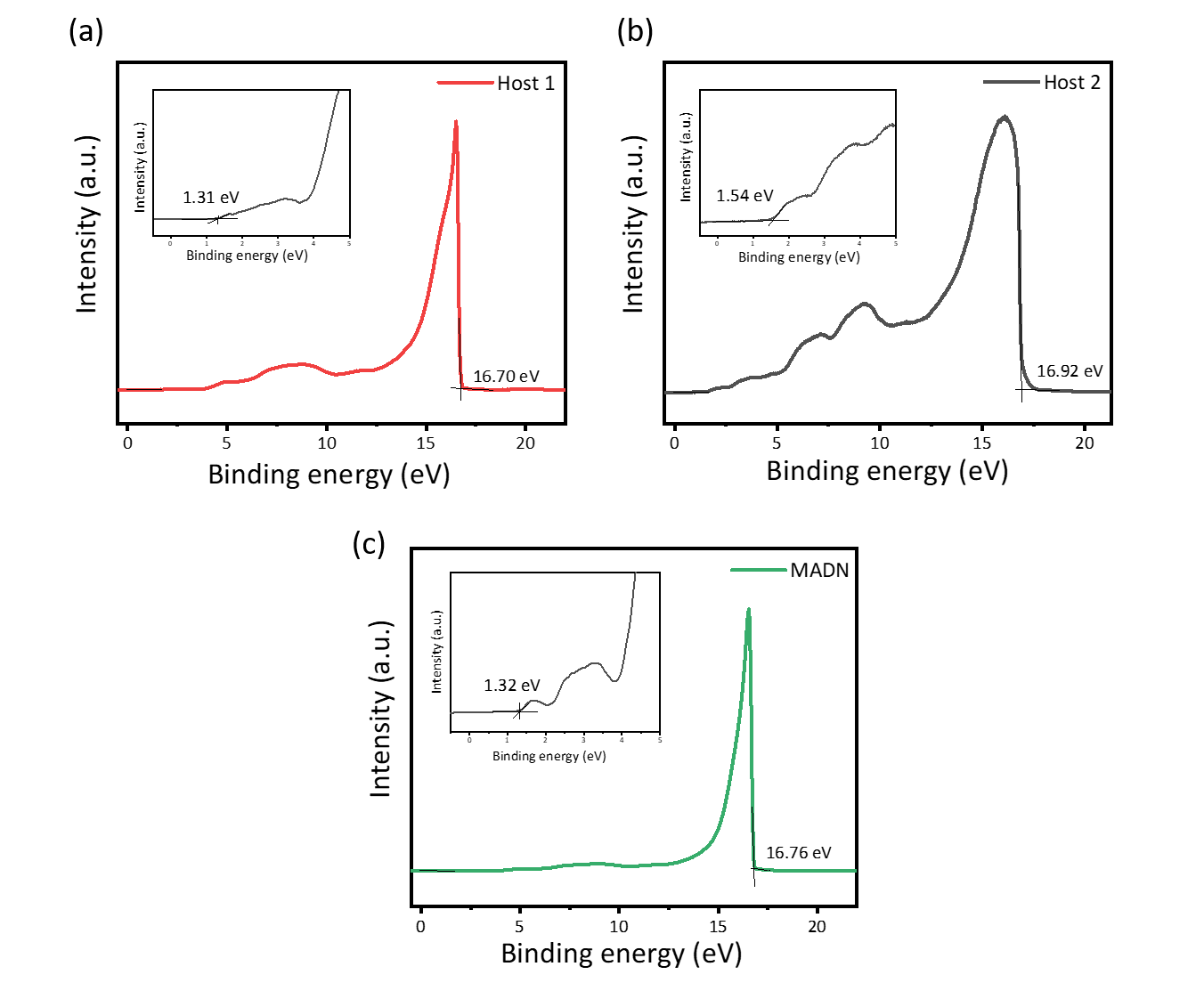


**Figure S21.** UPS spectra of host (a) **Host 1**, (b) **Host 2** and (c) MADN in film samples


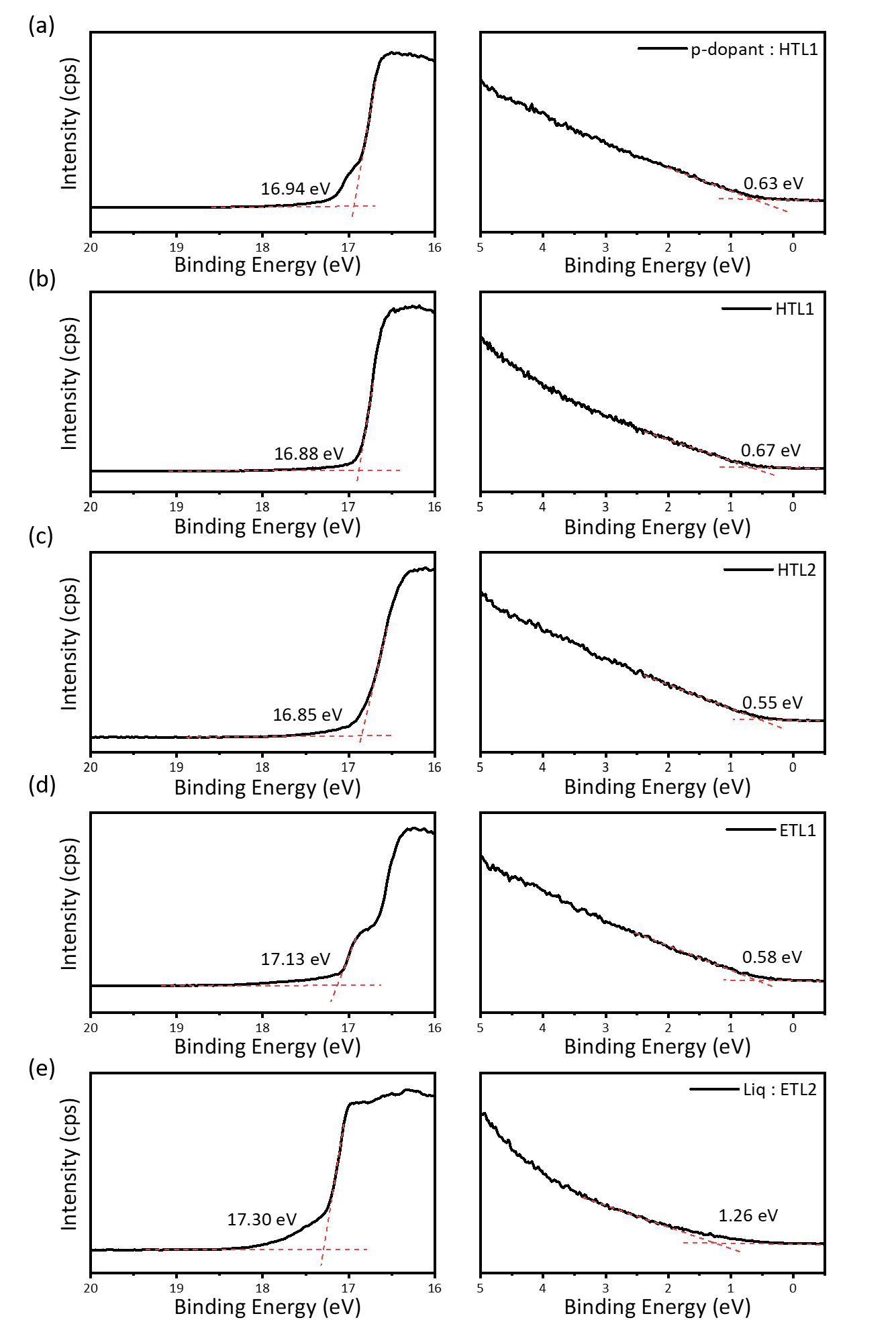


**Figure S22.** UPS spectra of organic layers in TTU device: (a) 3 wt% p-dopant: HTL1, (b)HTL1, (c) HTL2, (d) ETL1 and (e) 50 wt% Liq : ETL2 in film samples

**Table S15** The energy levels of organic layers in TTU device

| **Organic layers** | **HOMO^a^**  **[eV]** | **LUMO^b^**  **[eV]** |
| --- | --- | --- |
| **p-dopant: HTL1** | −4.91 | −1.83 |
| **HTL1** | −5.01 | −1.93 |
| **HTL2** | −4.92 | −1.87 |
| **ETL1** | −4.67 | −1.14 |
| **Liq: ETL2** | −5.18 | −2.27 |

^a^ Measured on film samples by ultraviolet photo-electron spectroscopy (UPS).

^b^ Estimated from the HOMO values and optical bandgap.

**Table S16**: Performance data of reported t-DABNA bottom-emitting OLED without sensitizer.

| **EML** | **EQE**  **[%]**  **Max/1000** | **EL Peak**  **/FWHM**  **[nm]** | **CIE**  **(x, y)** | **Device lifetime**  **(LT)** | **Ref.** |
| --- | --- | --- | --- | --- | --- |
| **mCPCz:**  **2% t-DABNA** | 18.0/5.2 | 462/25 | (0.13, 0.11) | T_80_=10 h  (At 1000 cd/m^2^) | ^[2]^ |
| **α,β-ADN:**  **2% t-DABNA** | 10.3/10.3 | 461/25 | (0.133, 0.085) | T_90_ =79.2 h  (At 2000 cd/m^2^) | ^[3]^ |
| **DPEPO:**  **1% t-DABNA** | 13.0/0.4 | 461/25 | (0.14, 0.07) | T_90_ < 1 h  (At 100 cd/m^2^) | ^[4]^ |
| **mCBP: mCBP-CN:**  **3% t-DABNA** | 25.2/- | 465/27 | (0.126, 0.102) | T_50_ < 2 h  (At 100 cd/m^2^) | ^[5]^ |
| **Host 1:**  **3% t-DABNA** | 11.7/11.5 | 463/24 | (0.125, 0.098) | T_90_ =249 h  (At 1000 cd/m^2^) | This work |

## 4. EQE-J curves fitting models

The singlet-polaron annihilation (SPA) model simulation can be described as follows^[6,7]^.

$\frac{\eta}{\eta_{0}}=\frac{1}{1+{(\frac{J}{J_{0}})}^{\frac{1}{l+1}}}$ Equation (1)

where η is the EQE in the presence of SPA, η_0_ is the external quantum efficiency without SPA (at very low current densities, rendering the SPA quenching negligible) and J_0_ is the characteristic current density when the initial efficiency drops by half.

Assuming bulk limited transport, the equation for triplet-polaron annihilation (TPA) model can beexpressed as follows^[8]^.

$\frac{\eta}{\eta_{0}}=\frac{1}{1+{CJ}^{\frac{1}{l+1}}}$ Equation (2)

where η_0_ is the device EQE in the absence of TPA, J stands for the current density of the device, and C is a constant which is related to the parameters like dielectric constant, carrier mobility, TPA rate constant and decay lifetime of system. l needs to be an integer greater than 1 to make this equation reasonable, and here l = 2 after fitting.

The TTA mode simulation can be described as follows^[9]^.

$\frac{\eta}{\eta_{0}}=\frac{J_{0}}{4J}\left( \sqrt{1+8\frac{J}{J_{0}}} -1 \right)$ Equation (3)

where η, η_0_, and J_0_ represent the EQE in the presence of triplet-triplet annihilation (TTA), initial EQE in the absence of TTA (at very low current densities, rendering the TTA quenching negligible), and the current density at the half-maximum of the EQE, respectively.

## 5. Transient EL data

**Table S17** Transient EL data of the TTU devices. The fitting parameters are derived
from TTU fitting model $y=\frac{1}{\left( At+B \right)^{2}}$ .

| Device | Voltage  (V) | A | B | Delayed ratio (%) |
| --- | --- | --- | --- | --- |
| 3 wt% **t-DABNA** in **Host 1** | 4 | 0.72 | 2.83 | 12.49 |
|  | 5 | 0.57 | 2.29 | 19.07 |
|  | 6 | 0.44 | 1.93 | 26.85 |
|  | 7 | 0.47 | 1.89 | 27.99 |
|  | 8 | 0.39 | 1.65 | 36.73 |
| 3 wt% **t-DABNA** in **Host 2** | 4 | 0.65 | 2.62 | 14.57 |
|  | 5 | 0.53 | 2.18 | 21.04 |
|  | 6 | 0.46 | 1.91 | 27.41 |
|  | 7 | 0.46 | 1.83 | 29.86 |
|  | 8 | 0.41 | 1.72 | 33.80 |
| 3 wt% **t-DABNA** in **MADN** | 4 | 0.56 | 2.72 | 13.52 |
|  | 5 | 0.50 | 2.35 | 18.11 |
|  | 6 | 0.52 | 2.30 | 18.90 |
|  | 7 | 0.43 | 1.99 | 25.25 |
|  | 8 | 0.37 | 1.77 | 31.92 |

## 6. Top-emitting device data

**Table S18.** Summarized top-emitting device characteristics.

| **Host in EML** | **V_on_^a^**  **[V]** | **η_ext_ [%]**  **Max** | **η_c_**  **[cd A^-1^]** | **C.E./y**  **[cd A^-1^]** | **λ_EL_/FWHM^b^**  **[nm]** | **CIE^b^**  **(x,y)** | **LT_90_^c^**  **[hrs]** | |
| --- | --- | --- | --- | --- | --- | --- | --- | --- |
| **Host 1** | 3.36 | 15.1 | 7.3 | 154.3 | 462/16 | (0.136, 0.048) | 74.5 | |
| **Host 2** | 3.36 | 13.2 | 5.9 | 137.3 | 460/16 | (0.139, 0.043) | 72.0 | |
| MADN | 3.62 | 13.0 | 6.3 | 136.6 | 461/16 | (0.137, 0.046) | 33.3 | |
| ^a^ Measured value at brightness >1 cd m^-2^; ^b^ Measured at 10 mA cm^-2^; ^c^ Initial Luminance of 1000 cd m^-2^. | | | | | | | |  |

## References

[1] D.U. Heo, J. Y. Choi, M. Han, J. T. Lee, J. M. Yoon, J. Lee, H. Yun, H. J. Kim, H. Park, S. K. Hong, W. Lee, Korean Patent No.[WO2022039365A1](https://worldwide.espacenet.com/patent/search?q=pn%3DWO2022039365A1), **2022**.

[2] D. D. Zhang, X. Z. Song, A. J. Gillett, B. H. Drummond, S. T. E. Jones, G. M. Li, H. Q. He, M. H. Cai, D. Credgington, L. Duan, *Adv. Mater.* **2020**, *32*,1908355.

[3] Z. Liu, X. Li, Y. Lu, C. Zhang, Y. Zhang, T. Huang, D. Zhang, L. Duan, *Nat. Commun*. **2022**, 13, 1215.

[4] S. H. Han, J. H. Jeong, J. W. Yoo, J. Y. Lee, *J. Mater. Chem. C* **2019**, *7*, 3082.

[5] J. Park, K. J. Kim, J. Lim, T. Kim, J. Y. Lee, *Adv. Mater.* **2022**, *34*, 2108581.

[6] K. Hayashi, H. Nakanotani, M. Inoue, K. Yoshida, O. Mikhnenko, T. Q. Nguyen, C. Adachi, *Appl. Phys. Lett.* **2015**, *106,* 093301.

[7] Y. Setoguchi, C. Adachi, *J. Appl. Phys.* **2010**, *108*, 064516

[8] X. L. Lv, Y. X. Wang, N. Q. Li, X. S. Cao, G. H. Xie, H. Huang, C. Zhong, L. Wang, C. L. Yang, *Chem. Eng. J.* **2020**, *402*, 126173.

[9] X. L. Lv, J. S. Miao, M. H. Liu, Q. Peng, C. Zhong, Y. X. Hu, X. S. Cao, H. Wu, Y. Y. Yang, C. J. Zhou, J. Z. Ma, Y. Zou, C. L. Yang, *Angew. Chem.* **2022**, *61*, 202201588.
